# The Novel, Nicotinic Alpha7 Receptor Partial Agonist, BMS-933043, Improves Cognition and Sensory Processing in Preclinical Models of Schizophrenia

**DOI:** 10.1371/journal.pone.0159996

**Published:** 2016-07-28

**Authors:** Linda J. Bristow, Amy E. Easton, Yu-Wen Li, Digavalli V. Sivarao, Regina Lidge, Kelli M. Jones, Debra Post-Munson, Christopher Daly, Nicholas J. Lodge, Lizbeth Gallagher, Thaddeus Molski, Richard Pieschl, Ping Chen, Adam Hendricson, Ryan Westphal, James Cook, Christiana Iwuagwu, Daniel Morgan, Yulia Benitex, Dalton King, John E. Macor, Robert Zaczek, Richard Olson

**Affiliations:** 1 Discovery Biology, Bristol-Myers Squibb Company, Wallingford, Connecticut, United States of America; 2 Lead Discovery and Optimization, Bristol-Myers Squibb Company, Wallingford, Connecticut, United States of America; 3 Discovery Chemistry, Bristol-Myers Squibb Company, Wallingford, Connecticut, United States of America; 4 Pharmaceutical Candidate Optimization, Bristol-Myers Squibb Company, Wallingford, Connecticut, United States of America; Chiba University Center for Forensic Mental Health, JAPAN

## Abstract

The development of alpha7 nicotinic acetylcholine receptor agonists is considered a promising approach for the treatment of cognitive symptoms in schizophrenia patients. In the present studies we characterized the novel agent, (2*R*)-*N*-(6-(1*H*-imidazol-1-yl)-4-pyrimidinyl)-4'*H*-spiro[4-azabicyclo[2.2.2]octane-2,5'-[1,3]oxazol]-2'-amine (BMS-933043), *in vitro* and in rodent models of schizophrenia-like deficits in cognition and sensory processing. BMS-933043 showed potent binding affinity to native rat (Ki = 3.3 nM) and recombinant human alpha7 nicotinic acetylcholine receptors (Ki = 8.1 nM) and agonist activity in a calcium fluorescence assay (EC50 = 23.4 nM) and whole cell voltage clamp electrophysiology (EC50 = 0.14 micromolar (rat) and 0.29 micromolar (human)). BMS-933043 exhibited a partial agonist profile relative to acetylcholine; the relative efficacy for net charge crossing the cell membrane was 67% and 78% at rat and human alpha7 nicotinic acetylcholine receptors respectively. BMS-933043 showed no agonist or antagonist activity at other nicotinic acetylcholine receptor subtypes and was at least 300 fold weaker at binding to and antagonizing human 5-HT3A receptors (Ki = 2,451 nM; IC50 = 8,066 nM). BMS-933043 treatment i) improved 24 hour novel object recognition memory in mice (0.1–10 mg/kg, sc), ii) reversed MK-801-induced deficits in Y maze performance in mice (1–10 mg/kg, sc) and set shift performance in rats (1–10 mg/kg, po) and iii) reduced the number of trials required to complete the extradimensional shift discrimination in neonatal PCP treated rats performing the intra-dimensional/extradimensional set shifting task (0.1–3 mg/kg, po). BMS-933043 also improved auditory gating (0.56–3 mg/kg, sc) and mismatch negativity (0.03–3 mg/kg, sc) in rats treated with S(+)ketamine or neonatal phencyclidine respectively. Given this favorable preclinical profile BMS-933043 was selected for further development to support clinical evaluation in humans.

## Introduction

Schizophrenia is a severe disorder affecting 0.5–1% of the population and resulting in poor social and occupational functioning. The clinical features are clustered into 3 symptom groups; i) positive symptoms (delusions, hallucinations, thought disorder and disorganized behavior), ii) negative symptoms (social withdrawal, avolition, affective disturbances, alogia and anhedonia) and iii) cognitive symptoms which include abnormalities in selective attention, working memory, executive function, episodic memory, language comprehension and social-emotional processing. While the emergence of positive symptoms in early adulthood is the most striking clinical feature, cognitive deficits are a core feature of the disorder, are present prior to the onset of psychosis and are the single best predictor of long term functional outcome [1–2]. Furthermore, while current antipsychotic drugs effectively manage positive symptoms in some patients, cognitive and negative symptoms are poorly treated and the identification of new therapeutic approaches remains a high priority [3–4].

While several novel approaches have progressed to clinical evaluation, agents that activate the alpha7 nicotinic acetylcholine receptor (α7 nAChR) have received considerable attention. Neuronal nAChRs are heterogeneous, ligand activated cation channels with a pentomeric structure consisting of five heteromeric or homomeric subunits arranged around a central cation pore [5–6]. Homomeric α7 nAChRs are notable for their high permeability to Ca^2+^, rapid desensitization, low affinity for nicotine and high affinity for the antagonist, methyllycaconitine (MLA) [7]. These receptors are highly expressed in the cortex and hippocampus and activation results in i) increased presynaptic release of gamma aminobutyric acid (GABA), glutamate, dopamine, acetylcholine and 5-hydroxytryptamine (5-HT), ii) increased postsynaptic cell excitability through direct membrane depolarization and iii) activation of intracellular, calcium-dependent, biochemical signaling cascades that are critical for synaptic strength, plasticity and the formation of long term memory [8–10]. Alpha7 nAChRs are also localized in the prefrontal cortical circuits thought to mediate the higher order cognitive deficits seen in schizophrenia patients [11]. These deficits have been attributed to decreased excitability of glutamatergic pyramidal neurons and defective synchronization of pyramidal network firing due to deficient GABA release from parvalbumin positive GABAergic interneurons [12]. Activation of α7 nAChRs on pyramidal cells, presynaptic glutamate terminals and parvalbumin positive GABAergic interneurons can potentially address both deficits by directly (via membrane depolarization) and indirectly (via glutamate and dopamine release) increasing pyramidal cell excitability and enhancing GABA release [13–15]. On the basis of these findings several agents with potent α7 nAChR agonist activity have been evaluated in preclinical models including tropisetron, one of the earliest agents identified with high affinity for this receptor [16]. While tropisetron is also a potent 5-HT_3_ receptor antagonist used clinically for the treatment of post-operative and chemotherapy induced nausea and emesis, this agent alleviates cognitive deficits in phencyclidine (PCP) treated mice, an effect which was blocked by MLA consistent with α7 nAChR involvement [17]. These findings have subsequently been extended with several novel α7 nAChR agonists reported to improve cognition in rodents treated with N-methyl-D-aspartate (NMDA) receptor antagonists to mimic schizophrenia-like deficits including RG3487 (N-[(3S)-1-azabicyclo[2.2.2]oct-3-yl]-1H-indazole-3-carboxamide hydrochloride), SSR-180711 (4-bromophenyl 1,4-diazabicyclo[3.2.2]nonane-4-carboxylate), A-582941 (octahydro-2-methyl-5-(6-phenyl-3-pyridazinyl)-pyrrolo[3,4-c]pyrrole dihydrochloride), TC-5619 ((*2S*,*3R*)-N-[2-(pyridin-3-ylmethyl)-1-azabicyclo[2.2.2]oct-3-yl]benzo[b]furan-2-carboxamide) and EVP-6124 ((*R*)-7-chloro-N-(quinuclidin-3-yl)benzo[b]thiophene-2-carboxamide) [18–24].

Treatment with α7 nAChR agonists may also alleviate the deficits in pre-attentive, sensory information processing which are also seen in schizophrenia patients. The successful processing of sensory inputs requires the ability to screen out or ‘gate’ intrinsic responses to redundant or irrelevant sensory stimuli and reciprocally, enhance or facilitate responses to deviant, novel or salient stimuli. Electroencephalogram based neurophysiological measures indicate that both aspects are impaired in schizophrenia patients as shown by reductions in P50 suppression (gating) and mismatch negativity (MMN; deviance detection) [25]. Evidence supporting an involvement of α7 nAChRs includes genetic linkage studies showing that P50 deficits are linked to chromosome 15q14, the site of the CHRNA7 gene which encodes the α7 nACh subunit protein [26]. Furthermore P50 gating can be improved after α7 nAChR agonist treatment in DBA/2 mice which have a natural gating deficit due to lower α7 nACh receptor expression [22, 27–28]. Finally improvements in P50 suppression and MMN have been reported in schizophrenia patients treated with tropisetron and DMXB-A (3-[(3*E*)-3-[(2,4-dimethoxyphenyl) methylidene]-5,6-dihydro-4*H*-pyridin-2-yl]pyridine) or EVP-6124 respectively [29–31].

The present studies describe the pharmacological characterization of BMS-933043 ((2*R*)-*N*-(6-(1*H*-imidazol-1-yl)-4-pyrimidinyl)-4'*H*-spiro[4-azabicyclo[2.2.2]octane-2,5'-[1,3]oxazol]-2'-amine) a novel, selective α7 nAChR partial agonist. The evaluation of BMS-933043 includes the demonstration of efficacy in preclinical cognition models, improvement of sensory processing measures with translational biomarker potential and demonstration of the relationship between efficacy, drug exposure and α7 nAChR occupancy. On the basis of these favorable preclinical results BMS-933043 was selected as a lead candidate and advanced into clinical development for the treatment of cognitive and negative symptoms of schizophrenia.

## Materials and Methods

### In Vitro Studies

#### Reagents

HEK293 cell lines expressing either the rat or human α7 nAChR (+ resistance to inhibitors of cholinesterase 3 (RIC-3)), rat α1β1δε nAChR, rat α4β2 nAChR, rat α3β4 nAChR or human 5-HT_3A_ receptor were developed by Bristol-Myers Squibb (Wallingford, CT, USA). The following chemicals were synthesized by Bristol-Myers Squibb: A-582941, BMS-933043, EVP-6124, NS-6740 (4-[[5-[3-(trifluoromethyl)phenyl]-2-furanyl]carbonyl]-1,4-diazabicyclo[3.2.2]nonane), PNU-282987 (N-(*3R*)-1-azabicyclo[2.2.2]oct-3-yl-4-chloro-benzamide hydrochloride), TC-5619, [^3^H]-A585539 (^3^H-2,2-dimethyl-5-(6-phenyl-3-pyridazinyl)-5-aza-2-azoniabicyclo[2.2.1]heptane iodide; specific activity 71 Ci/mmol, concentration 1mCi/ml). Suppliers for other reagents were as follows: (±)epibatidine dihydrochloride hydrate (FLIPR studies), phencyclidine hydrochloride (PCP), (+)MK-801 hydrogen maleate ([5R,10S]-[+]-5-methyl-10,11-dihydro-5*H*-dibenzo[*a*,*d*]cyclohepten-5,10-imine), S(+)ketamine HCl and (-)nicotine hydrogen tartrate salt (Sigma-Aldrich, Saint Louis, Mo); methyllycaconitine citrate, (±)epibatidine (binding studies) and MDL-72222 (tropanyl 3,5-dichlorobenzoate BioTechne, Minneapolis, MN); [^3^H]granisetron ([^3^H]BRL-43694, specific activity 83.66 Ci/mmol, concentration 1 mCi/ml; Perkin Elmer, Waltham, MA).

#### Calcium flux assays

Functional activity was examined using Ca^2+^-sensitive, dye-based, microfluorometric assays read on the FLIPR Tetra plate reader platform (Molecular Devices, CA, USA). Cells were plated in 384-well black, clear-bottomed Greiner poly-D-lysine coated plates at a density of ~25 K cells/well and incubated at 29°C for 48 h. Prior to assay, media contents were removed and cells incubated in assay buffer containing 5μM final Fluo-4 dye for 90 min. For nAChR assays, the dye-loading buffer was HBSS (#14175 Life Technologies) supplemented with 20 mM HEPES, 0.5 mM CaCl_2_, 1 mM MgCl_2_ and 10 μM atropine (final concentration). Test compounds were solubilized in DMSO and serially diluted in NUNC 384-well plates prior to addition of assay buffer (5.3 mM KCl + 140 mM NaCl + 20 mM Hepes + 25 mM CaCl_2_ + 1 mM MgCl_2_ + 10 μM atropine final concentration). Compounds diluted in assay buffer were added to the assay plate by the FLIPR and read for approximately 2 min. Increases in fluorescence induced by agonist activity of test compounds were normalized to the activity of an EC_100_ concentration of ACh (30 μM for α1β1δε and α4β2; 300 μM for α7 and α3β4). For antagonist studies FLIPR assays were conducted as described above except that cells were pre-incubated with test agent for 30 min prior to the addition of an EC_90_ concentration of ACh (10 μM for α1β1δε and α4β2; 150 μM for α3β4) and the fluorescence signal normalized to the ACh response alone. For 5-HT_3A_ antagonist studies, the dye loading and compound diluent buffer was HBSS (#14025 Life Technologies) supplemented with 20 mM HEPES (final concentration) and the concentration of 5-HT used in the assay was 2 μM. Offline transformation of concentration response data was done using a 4 parameter logistic fit using a Bristol-Myers Squibb proprietary informatics software suite.

#### Radioligand binding assays

Radioligand binding assays were performed on membrane homogenates prepared from whole rat brain (minus cerebellum), HEK293/rat α7 nAChR cells or HEK293/human 5-HT_3A_ cells. Membranes were prepared by homogenizing samples at 4°C in hypotonic lysis buffer consisting of 10 mM Tris (pH 7.4), 5 mM EDTA and protease inhibitors followed by centrifugation at 32,000 x g for 20 min. This step was repeated and the resulting pellet re-suspended in buffer containing either i) 50 mM KH_2_PO_4_ (pH 7.4 @ 25°C), 1 mM EDTA, 0.005% Triton-X 100 and 0.1% (v/v) Sigma Protease Inhibitor Cocktail for α7 nACh binding assays or ii) 140 mM NaCl, 2.8 mM KCl, 1.0 mM CaCl_2_, 2.0 mM MgCl_2_ and 10 mM HEPES (pH 7.4 @ 37°C) for 5-HT_3A_ binding assays. Protein concentrations were determined using methods described in [32] with bovine serum albumin as a standard and aliquots were then frozen in dry ice/ethanol and kept at -80°C until the day of the assay. Binding to α7 nAChR was determined using [^3^H]-A-585539 filtration binding as described in [33] with minor modification. Frozen aliquots of membrane homogenate were thawed, homogenized and re-suspended at 75 μg/well protein in assay buffer (120 mM NaCl, 5 mM KCl, 2 mM CaCl_2_, 2 mM MgCl_2_ and 50 mM Tris-Cl, pH 7.4, 4°C). In saturation binding experiments, the membrane preparation was incubated at 4°C for 90 min in the presence of increasing concentrations of [^3^H]-A-585539. Non-specific binding was defined with 10 μM MLA. Competition binding experiments were performed using a single concentration of [^3^H]-A-585539 (0.5 nM) in the presence of 5 increasing concentrations (in duplicate) of test compound.

Binding to 5-HT_3A_ receptors was determined using [^3^H]-Granisetron filtration binding performed as described in [34] with minor modification. Frozen aliquots of membrane homogenate were thawed, homogenized and re-suspended at 6.6 μg/well protein in 5-HT_3A_ binding assay buffer. In saturation binding experiments, the membrane preparation was incubated at 37°C for 60 min in the presence of increasing concentrations of [^3^H]-Granisetron. Non-specific binding was defined with 10 μM MDL-72222. Competition binding experiments were performed using a single concentration of [^3^H]-Granisetron (1 nM) in the presence of 5 increasing concentrations (in duplicate) of test compound. For both assays the reaction was terminated by the addition of 5 ml of ice-cold assay buffer and rapid filtration through a Brandel Cell Harvester using Whatman GF/B filters presoaked in 0.3% polyethylenimine. The filter was then punched onto a 96 well microbeta sample plate, 200 μl/well of Packard Ultima Gold XR scintillation fluid added, soaked overnight and then counted in a LKB Trilux liquid scintillation counter. IC50 values were determined using non-linear regression four-parameter logistic equation, y = A + ((B-A)/(1+((C/x)^D))) where A = 0% inhibition, B = 100% inhibition, C = log IC_50_ and D = slope factor (Graphpad Prism version 5.01). K_i_ (apparent) values were calculated for competitor compounds using the method of Cheng and Prusoff.

#### Whole cell patch clamp electrophysiology

Whole-cell voltage clamp studies were conducted on HEK293/rat α7 or HEK293/human α7 nAChR cells. Cells were plated at a density of 125K/35 mm dish on uncoated glass coverslips. The external bathing solution consisted of: 120 mM NaCl, 3 mM KCl, 2 mM CaCl_2_, 2 mM MgCl_2_, 10 mM HEPES, 25 mM glucose, pH 7.4 with TRISBase, 270–275 mOsm. Atropine (1 μM) was included in the bathing solution. The internal pipette solution contained: 110 mM TRIS phosphate dibasic, 11 mM EGTA, 2 mM MgCl_2_, 0.1 mM CaCl_2_, 4 mM Na2ATP, pH 7.3 with TRISBase (20.4 mM), 265–270 mOsm. Cells were voltage clamped at a holding potential of -90 mV. Using the Dynaflow microfluidic perfusion system (Cellectricon; flow 26 μl/min) responses to a 300 ms application of ACh or α7 nAChR agonists were measured. Currents were filtered at 2 kHz and sampled at 10 kHz using an EPC-9 amplifier and HEKA Pulse/Patchmaster software. For BMS-933043, a 1 min wash with buffer was sufficient to allow the α7 nAChR to fully recover from desensitization allowing repeated BMS-933043 applications per cell. In contrast, full recovery was not achieved following application of EVP-6124 or TC-5619 requiring longer wash periods between successive applications (EVP-6124) or testing single concentrations per cell (TC-5619) to generate the concentration response curve. The peak current amplitude and net charge crossing the membrane (i.e. the area under the current response curve) induced by agonist application were measured using Clampfit (pClamp Suite) and normalized to the maximal response induced by ACh (3 mM ACh for peak current amplitude and 300 μM ACh for net charge). The normalized maximum peak current is presented as the Ymax_obs_ @ 10 μM and the EC_50_ and Ymax values for net charge crossing the membrane were calculated using Excel-XLFit. To examine inhibition of the BMS-933043 response, HEK293/rat α7 nAChR cells were pre-incubated with the antagonist MLA (10 nM) or the silent agonist NS-6740 (10–3000 nM) for 1 or 2 min prior to the application of BMS-933043 (10 μM) and current responses normalized to the response induced by BMS-933043 alone.

### In Vivo Studies

#### Animals

All in vivo studies were carried out in strict accordance with the recommendations in the Guide for the Care and Use of Laboratory Animals of the National Institutes of Health. Study protocols were approved by the Animal Care and Use Committee of the Bristol-Myers Squibb Company. Studies were conducted in male C57Bl/6 mice (25–30g; Taconic Laboratories, New York), male Sprague Dawley rats (+ maze set shift task: 150–175g at delivery (7–11 weeks of age at testing), Harlan, Dublin, VA; MMN and N40 gating: 200–300g at delivery, Harlan, Indianapolis, IN) or male offspring from timed pregnant Sprague Dawley rats (gestational day 10–13 at delivery; Harlan, Indianapolis, IN). Animals were held in colony rooms maintained at constant temperature (21 ± 2°C) and humidity (50 ± 10%) and illuminated for 12 h per day (lights on at 0600 h). Animals were group housed (n = 3–4/cage) and had *ad libitum* access to food and water unless specified otherwise. On completion of studies subjects were euthanized by either CO_2_ inhalation or rapid decapitation for occupancy and/or drug exposure determinations. Behavioral studies were conducted in either mice or rats and carried out between 0600 and 1300 h. The novel object recognition test was used as the primary in vivo screening assay to identify novel α7 nAChR agonists and was conducted in mice because of the small drug quantities required for testing in this species. For attentional set shifting paradigms rats were selected because of prior literature establishing a key role for the medial prefrontal cortex (mPFC) in this species [35–36] and the ability of NMDA antagonist treatment to impair performance in adults and neonatal subjects [19, 37–38]. Finally we examined a Y maze task using a modified continuous performance procedure previously described in mice and reported to show improvement after α7 nAChR agonist treatment in this species [39].

All surgical procedures were performed under isoflurane anesthesia (5% induction, 1–4% maintenance) and conducted using aseptic technique. Core temperature was monitored and maintained between 36 ± 1°C and the rate and depth of respiration monitored throughout the procedure. Local anesthetic (lidocaine ointment, 5%) was applied to the ear via the atraumatic ear bars and after placement in the stereotaxic frame the head and dorsal neck regions were clipped and scrubbed with 70% alcohol followed by 10% betadine solution. Following surgery skin incisions were closed in layers using sterile sutures and subjects were treated with buprenorphine (0.05 mg/kg, sc) for postoperative analgesia and monitored until complete recovery from anesthesia. All subjects received azithromycin (10 mg/kg, po) once daily for up to 10 days and were housed singly with standard food and water available *ad libitum*. Animals were given adequate recovery time from surgery and were acclimated to the EEG recording chambers/procedure over weeks before being enrolled in studies.

#### Mouse 24 h novel object recognition procedure

The procedure followed a 3 day protocol. On day 1 (habituation) individual mice were habituated to the testing chamber (a white PVC box 48 cm long x 38 cm wide x 20 cm high with brown linoleum flooring) for 15 min then returned to their home cage. On day 2 (training) individual mice were returned to the testing chamber containing 2 identical objects and allowed to explore. The training session lasted for up to 15 min or until the mouse explored the objects for a total of 60 s. A maximal trial duration of 15 min was used to accommodate mice that are slower to initiate exploration. On day 3 (testing) individual mice were returned to the testing chamber 24 h later containing 1 familiar object encountered in the previous training session and one novel object. The testing session lasted up to 10 min or until the mouse explored the objects for a total of 60 s. The objects used for these studies were made of duplex legos approximately 3 inches high x 1 inch wide; one object was a multicolored rectangle and the second object was a solid color rectangle with a short arm attached. The location of the objects was counterbalanced between animals but was consistent within animals so that only the object itself was novel on the test session. The familiar object used during the test session was the same object used during training and all objects were thoroughly cleaned between sessions and between subjects. Animal behavior was video recorded during both training and testing and the amount of time spent exploring the objects determined using Cleversys software. Object exploration was only scored when the animal’s nose was within 1 cm of the object. Subjects were dosed subcutaneously (sc) with either BMS-933043 or vehicle (0.9% saline, pH4; 10 ml/kg) 30 min prior to the training session only and tested for recognition memory 24 h later. The vehicle was chosen for sc dosing to ensure that BMS-933043 was dosed as a solution and not a suspension. To examine inhibition of the BMS-933043 response subjects were treated with NS-6740 (10 mg/kg sc) 10 min prior to BMS-933043 (0.3 mg/kg, sc) with the training session commencing 30 min later as above. For each subject the % discrimination index (DI) was calculated for both the training and test session as follows: %DI training = (time exploring object 1—time exploring object 2)/(total time exploring object 1 + object 2) X 100; %DI testing = (time exploring novel object—time exploring familiar object)/(total time exploring novel + familiar object) X 100.

#### Rat MK-801 set shift procedure

Studies followed the methods described in detail in [19] and are briefly summarized here. All rats were singly housed and food-restricted (15g/rat/day) for 7 days prior to initiation of training and for the duration of the study. At the time of testing, the average (± SEM) body weight for individual treatment groups ranged from 168 ± 4 g to 180 ± 6 g. Prior to the study several habituation sessions were conducted to familiarize subjects to the maze and task requirements. The maze consisted of 4 arms (40 cm long x 14 cm wide x 20 cm high) arranged in a ‘+’ configuration and the walls and floor of each arm were distinct and colored either black or white with either a rough or smooth texture (i.e. 1 arm with each of the following combinations: white/smooth, white/rough, black/smooth, black/rough). In the final stages of training and during the set shift procedure the maze was set up in a ‘T’ configuration with 1 arm completely blocked. The arm opposite the blocked arm was designated as the ‘start arm’ and only 1 of the 2 remaining arms was baited with food pellets. The set shift procedure comprised 2 test sessions conducted over 2 consecutive days. On the first day (Set 1) rats were required to learn the association of either the color or texture of the maze arms with the location of a food reward. Animals were randomly assigned a correct exemplar within a given dimension as being predictive of reward with 50% of the rats assigned color and 50% assigned texture. Each rat was given a maximum of 120 trials on the maze which was rotated 90 degrees between successive trials so that the adjacent arms now became either the ‘start arm’ or the arm that was blocked. Animals were allowed to visit 1 of the 2 open arms and the trial recorded as correct if the arm with the pre-assigned exemplar was visited and the food reward retrieved. The inter-trial interval was 15 s. Rats were required to achieve a performance criterion of 8 consecutive entries into the rewarded arm at which point testing stopped and the animal was presumed to have learnt the association between the correct exemplar in the assigned dimension and food reward. On day 2 (Set 2) animals were again tested in the maze configured as described for Set 1 with the exception that the dimension predicting food reward was now switched (i.e. animals trained on color for Set 1 were switched to texture and visa versa). Correct performance was again defined as 8 consecutive entries into the rewarded arm with a maximal number of 80 trials allowed. All dosing was administered on day 2 prior to Set 2 testing. Subjects were dosed po with either BMS-933043 or vehicle (0.9% saline, pH 4.0; 2 ml/kg) 55 min prior to treatment with MK-801 (0.03 mg/kg, intraperitoneal (ip)) or saline (1 ml/kg, ip) and behavioral testing initiated 35 min later (i.e. 90 min post-treatment with BMS-933043). Results are presented as i) the number of trials to achieve performance criteria and ii) the number of perseverative errors i.e. a choice that was correct on Set 1.

#### Mouse MK-801 two trial Y maze procedure

Testing was conducted in a grey polyethylene plastic Y maze that had 3 arms, 2 of 40 cm length (test arms) and 1 of 20 cm length (start arm). Each arm had distinct optical cues covering the walls and a removable partition to occlude the arm as needed. The test consisted of a forced choice trial followed by a free-choice trial. For the forced choice trial the start arm and one test arm was open with access to the 2nd test arm blocked by the partition. Individual subjects were placed in the start arm and allowed to explore the open test arm for 8 min after which they were removed from the maze, placed in a holding cage and the maze was cleaned. Animals were then immediately placed back on the Y maze for the free choice trial and allowed to explore both open test arms for 4 min. The protocol was thus designed for minimal delay between the forced choice and free choice trials. Animal behavior was video recorded during both trials and the time spent in the previously accessible arm (ie familiar arm) and the previously blocked arm (ie novel arm) determined during the free choice trial using Cleversys software. Subjects were dosed sc with either BMS-933043 or vehicle (0.9% saline, pH4; 10 ml/kg) 5 min prior to dosing with either saline (10 ml/kg, sc) or MK-801 (0.05 mg/kg, sc) and then tested in the 2 trial task 30 min later. For each subject the % time exploring the novel arm during the free choice trial was calculated using the formula: % novel time = novel time/(novel + familiar time) x 100.

#### Rat neonatal PCP intra-dimensional/extra-dimensional (ID/ED) procedure

Male pups were treated with either 0.9% saline (sham) or PCP (20 mg/kg, sc) on post-natal days 7, 9 and 11 and then singly housed after weaning on postnatal days 21–25. All studies were conducted in adult animals (2–4 months of age) using methods adapted from [35]. Rats were food-restricted (15g/rat/day) for 7 days prior to the initiation of training and throughout the remainder of the study. At the time of testing the average (± SEM) body weight ranged from 278 ± 6g to 296 ± 9g across the individual treatment groups. The animals then received 3 days of training during which they were handled, habituated to the test arena (5 min/day) and trained to dig in a ceramic pot for a food reward (Honey Nut Cheerios). The testing arena was constructed of black plastic (45 cm wide x 60 cm long x 45 cm tall) and divided into 3 compartments, i.e. a separate starting box and 2 choice boxes separated by an opaque wall. Each choice box contained a terracotta (4 inch diameter) pot, filled with digging media and with scented oil applied to the rim. At the beginning of each trial the animal was placed in the start box, the sliding door was opened and the subject was allowed to freely explore the two choice boxes. Both pots were initially baited with food until the subject reliably retrieved the food rewards from each. Thereafter, rats were trained to discriminate between either 2 distinct odors (stimulus dimension 1) or 2 distinct digging media (stimulus dimension 2) in order to correctly identify the baited pot. On the first trial only, rats were allowed to dig in both pots if an error was made (with the trial scored as an error trial). Thereafter animals were allowed to dig in one pot only per trial and were tested until the performance criterion of 6 consecutive correct choices was achieved. Each trial was limited to a maximum duration of 5 min and any subject not achieving performance criteria was eliminated from the study. On completion of a successful odor discrimination animals were then trained to successfully complete a simple discrimination trial based on digging media. Odor and digging media used in these initial training trials were not used again for ID/ED testing. On the day of ID/ED testing rats were dosed orally (po) with either vehicle (distilled water; 1 ml/kg) or BMS-933043 90 min prior to testing. All subjects were brought to the experimental laboratory at least 30 min prior to behavioral assessment. To determine ID/ED performance rats were tested for their ability to successfully complete a series of discriminations based on either odor or digging media. In all instances subjects were pre-assigned one stimulus dimension (i.e. digging media or odor) as the correct predictor of the food reward for the first series of discriminations. Only at the final stage i.e. the extra-dimensional shift (EDS) was the subject required to shift attention away from the assigned stimulus dimension to a different dimension (e.g. odor to digging media) to correctly identify the baited pot. The sequence of discriminations was a follows: simple discrimination (SD), compound discrimination (CD), intra-dimensional shift 1 (IDS1), intra-dimensional shift 2 (IDS2), intra-dimensional shift 2 reversal (IDS2rev) and EDS and examples of exemplars are shown in S1 Table. For all discrimination stages successful completion was defined as 6 consecutive correct trials at which point subjects progressed to the next phase of the task. The test session ended when either i) all discrimination phases were successfully completed or ii) testing had proceeded for 120 min. The average duration of a test session was 1 h and all subjects completed the ID/ED task within the allotted timeframe.

#### Rat neonatal PCP mismatch negativity procedure

Adult male rats treated neonatally with PCP were implanted with epidural screw electrodes at the following co-ordinates under isoflurane anesthesia: 6 mm rostral and 1 mm off midline to the left from bregma; 5.5 mm rostral to interaural line and 1 mm off to the right from midline. Following recovery EEG recordings were collected from conscious rats housed in individual sound attenuated chambers (Med Associates, St Albans, VT) with the recording electrodes tethered through a low torque commutator system (Plastics One Inc, Roannoke, VA) to a differential amplifier (gain x1000; Warner Instruments, Hamden, CT). Data were digitally acquired using CED Power 1401 acquisition hardware (CED, Cambridge, UK). Baseline EEG was first recorded using Spike v5.19 or later software (CED, Cambridge, UK) to ensure electrical connectivity and an acceptable EEG signal. A simultaneous video was used to assess the general state of the animals during the recordings. MMN was evoked by the presentation of ‘deviant’ tones of 1.5 KHz, 50 ms duration presented at the rate of 12% of the standard, 1KHz, 50 ms tones. Tones (70 dB) were delivered through a speaker mounted in the top center of the housing chamber and presented at 450 ms intervals. In each experimental session 700 standard and 100 deviant tones were presented. Data were analyzed using Signal v4.09 software (CED, Cambridge, UK). Artifact free trials were visually identified, corrected for baseline shift using the first 100 ms of the pre-stimulus baseline, 30 Hz low-pass filtered using a second order Butterworth digital filter and averaged to give evoked potential for each subject. The average standard evoked potential was subtracted from the average deviant evoked potential to give a difference wave (i.e. MMN) for each rat. An example of the group mean evoked response potential following vehicle or BMS-933043 (1 mg/kg, sc) treatment is shown in S1 Fig. To quantify MMN, the area under the curve (AUC) from 150–120 msec (ie 50–100 msec after tone presentation) was calculated from the average difference wave for each subject. Each study had 3 treatment arms with all rats receiving all treatments sc in a cross-over design with at least 3 days between dosing.

#### Rat S(+)ketamine N40 gating procedure

Rats were stereotaxically implanted with a hippocampal recording electrode (from bregma: 4.5 mm caudal, 4.2 mm lateral, 4.0 mm ventral) and a cerebellar reference screw (from bregma: 11.0 mm caudal, 2.5 mm lateral) under isoflurane anesthesia. All subjects were allowed at least 10 days to recover and were singly housed for the remainder of the study. EEG recordings were collected from conscious rats as described above except that a two tone stimulus pair (5 dB SPL, 0.01 s duration, 0.5 s inter-stimulus interval, 1 KHz carrier frequency) was presented at an inter-trial interval of 10 s. One hundred fifty frames of 1 s EEG epochs encompassing the stimulus-evoked response were recorded and artifact free frames subsequently averaged using Signal 4.05 or later software (CED, Cambridge, UK). The evoked potential complex was visually identified as a peak that typically occurs between 20–30 ms post stimulus (‘P20’) and a trough that is prominent between 30–60 ms (‘N40’) post-stimulus onset, with the difference between these 2 landmarks measured as the evoked potential amplitude. The amplitude of the second tone-evoked potential (S2) was divided by the amplitude of the first tone-evoked potential (S1) to determine the gating ratio (S2/S1). Rats were assigned to 2 treatments namely vehicle (0.9% saline, pH 4; 2 ml/kg) or a specified dose of BMS-933043 (0.3, 0.56, 1, 3 or 10 mg/kg, sc) and placed into the sound attenuated chambers immediately after dosing. A 5 min EEG was monitored for a noise-free signal after which the baseline gating ratio for 150 tone pair trials was determined. Animals were then treated with S(+)ketamine (10 mg/kg, sc) and the gating ratio to an additional 150 tone pairs determined as described above. Thus each dose of BMS-933043 was tested with its own vehicle control condition using a cross-over design with at least 1 week between treatments and was considered a discrete experiment.

#### Statistical analysis

For mouse NOR studies the % DI was analyzed by one way analysis of variance (ANOVA) followed by Dunnett’s t test comparing all groups to vehicle or vehicle/BMS-933043 treated mice (Graphpad Prism v5.01). In addition, %DI results for the training session were also analyzed by 1 sample t test to compare each treatment group to a hypothetical mean value of zero (Graphpad Prism v5.01). For rat MK-801 set shift studies the number of trials to achieve performance criteria and the number of perseverative errors were analyzed by one way ANOVA followed by Dunnett’s post hoc analysis comparing all groups to vehicle/MK-801 treated rats (Graphpad Prism v5.01). For mouse MK-801 Y maze studies the % novel time was analyzed by one-way ANOVA followed by Dunnett’s post-hoc test comparing all groups to vehicle/MK-801 treated mice (Graphpad Prism v5.01). For rat S(+)ketamine N40 gating studies each dose of BMS-933043 was tested alongside a vehicle control treatment using a within subjects design and was considered an independent experiment. The gating ratio (S2/S1) was analyzed by 2 way repeated measures ANOVA followed by Sidak’s multiple comparison test (Graphpad Prism v7). For rat ID/ED studies the number of trials to criteria for all discriminations were analyzed by 2 way repeated measures ANOVA followed by Dunnett’s post hoc analysis comparing all groups to neonatal PCP/vehicle treated rats (Graphpad Prism v7). For rat neonatal PCP MMN studies the area under the curve (AUC) from 150–120 msec (ie 50–100 msec after tone presentation) was calculated from the average difference wave for each subject. A one sample, two-tailed t test was used to determine significant deviance from a hypothetical 0 for each treatment condition (Graphpad Prism v5.01).

#### Ex vivo α7 nACh receptor occupancy

Occupancy determinations were made either on completion of behavioral testing or in satellite groups of animals dosed in parallel with behavioral testing. At specified time points after dosing, the forebrain (for occupancy), the remaining hindbrain (for brain drug exposure) and plasma were collected, frozen in chilled isopentane and stored at -80°C until assayed. One forebrain hemisphere from each animal was thawed and homogenized in assay buffer (50 mM Tris-HCl, 120 mM NaCl, 5 mM KCl, 2 mM MgCl_2_ and 2 mM CaCl_2_) with the volume proportional to the weight of the tissue (53 mg tissue/ml buffer). Homogenized tissue aliquots were frozen immediately and stored at -80°C. On the day of the assay, frozen sample aliquots were thawed, needle homogenized and 1 mg of the brain tissue incubated for 5 min with 0.5–1 nM [^3^H]-A-585539 at 4°C in a 96 well plate. Non-specific binding was defined by adding 25 μM MLA to wells containing tissues from vehicle-treated animals. After incubation, the reactions were terminated by the addition of ice-cold assay buffer and rapid filtration through a Brandel Cell Harvester using FPXLR-196 filters. The filters were washed with ice-cold assay buffer, punched into a clear plate and 200 μl scintillation fluid added per well. Bound radioactivity was measured using a Wallac Microbeta liquid scintillation counter. Specific binding was calculated by subtracting the value of the non-specific binding from that of the total binding in each sample. The percent occupancy was calculated as [1 –(specific binding in drug treated mice/specific binding in vehicle treated mice)] x 100. Plots of α7 nAChR occupancy versus plasma or brain concentrations of BMS-933043 were fitted to a one-site binding model using nonlinear regression according to the following equation: % Occupancy = Bmax * C/ (EC_50_ + C) where Bmax is the maximal binding, C is the drug concentration, and EC_50_ is the concentration required for 50% receptor occupancy.

#### Plasma concentration analysis

Concentrations of BMS-933043 in plasma were determined using an LC-MS/MS assay. In brief, 50 μL of the plasma sample was extracted by protein precipitation with 150 μL acetonitrile containing an internal standard and 5 μL of supernatant injected into the LC-MS/MS system. Analytes were separated by reversed phase chromatography and detection of each analyte was achieved through selected reaction monitoring mode of mass spectrometry. Samples were quantified by calculated peak area ratio of analyte and internal standard against the calibration curve of BMS-933043 in plasma (0.198–813 ng/mL).

## Results

**Fig 1:**
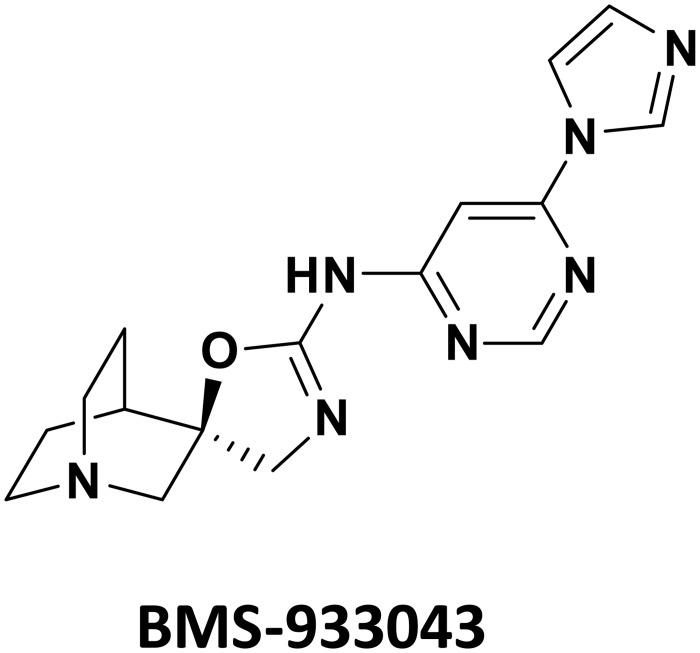
Molecular structure of BMS-933043.

BMS-933043 (2*R*)-*N*-(6-(1*H*-imidazol-1-yl)-4-pyrimidinyl)-4'*H*-spiro[4-azabicyclo[2.2.2]octane-2,5'-[1,3]oxazol]-2'-amine) has a molecular weight of 325.37 g/mol. The chemical structure of BMS-933043 is shown in Fig 1.

### Selective α7 nAChR Activation in Ca^2+^ Flux (FLIPR) Assays

Application of BMS-933043 to HEK293/rat α7 nAChR cells produced a concentration-dependent increase in fluorescence with a mean EC_50_ of 23.4 nM (Table 1). BMS-933043 did not exhibit agonist activity (EC_50_ >100 μM) or antagonist activity (IC_50_ >30 μM) at rat α1β1δε, α3β4 or α4β2 nAChR subtypes. Selective activation of α7 nAChRs was also observed following application of EVP-6124, TC-5619, A-582941 and PNU-282987 (Table 1) with negligible agonist activity detected at the other nAChR subtypes examined (EC_50_ >100 μM for all agents). In contrast, NS-6740 showed no α7 nAChR agonist activity whereas epibatidine showed potent agonist activity (EC_50_ ± SD) at α4β2 (4 ± 6 nM; n = 148), α3β4 (12 ± 5 nM, n = 153) and α7 nAChRs (53.2 nM ± 23.7; n = 289) but was weaker at the α1β1δε subtype (EC_50_ >1 μM, n = 150).

**Table 1:** Functional efficacy at recombinant human α7 nACh and 5-HT_3A_ receptors determined by Ca^2+^ flux (FLIPR) assays.

Agent	HEK293/Human α7 nAChR Mean ± SD EC_50_ (nM)	HEK293/Human 5-HT_3A_ Mean ± SD IC_50_ (nM)	Ratio
**BMS-933043**	23.4 ± 10.6 (n = 16)	8,066 ± 2,306 (n = 10)	345
**EVP-6124**	14.7 ± 11 (n = 4)	4.0 ± 2.1 (n = 5)	0.3
**TC-5619**	3.9 ± 1.9 (n = 5)	>100,000 (n = 9)	>25,641
**A-582941**	89.8 ± 54.3 (n = 6)	102 ± 86 (n = 10)	1.1
**PNU-282987**	94.5 ± 27 (n = 9)	24,920 ± 12,050 (n = 5)	264
**NS-6740**	>100,000 (n = 7)	4,827 ± 1,316 (n = 9)	Not calculated

### Binding Affinity at α7 nAChR

Saturation binding experiments showed that [^3^H]-A-585539 labeled a single population of receptors with a mean Kd of 152 pM and 656 pM at rat brain and recombinant human α7 nAChRs respectively (n = 2 determinations). Specific [^3^H]A-585539 binding was inhibited following incubation with BMS-933043, epibatidine, TC-5619, EVP-6124, A-582941, MLA, NS-6740 or (-)nicotine (Table 2; S2 Fig).

**Table 2:** Displacement of [^3^H]-A-585539 binding to rat brain or recombinant human α7 nAChR.

Agent	Rat Brain α7 nAChR Mean ± SD Ki (nM)	HEK293/Human α7 nAChR Mean ± SD Ki (nM)
**BMS-933043**	3.3 ± 0.45 (n = 6)	8.1 ± 1.96 (n = 4)
**Epibatidine**	5.2 ± 0.78 (n = 6)	10.5 ± 1.58 (n = 4)
**TC-5619**	0.23 ± 0.07 (n = 2)	0.9 ± 0.14 (n = 2)
**EVP-6124**	0.8 ± 0.33 (n = 2)	3 ± 1.13 (n = 2)
**A-582941**	21.7 ± 1.9 (n = 2)	82.6 ± 14.3 (n = 3)
**MLA**	0.25 ± 0.07 (n = 2)	4.17 ± 0.23 (n = 3)
**NS-6740**	2.85 ± 0.44 (n = 3)	Not tested
**(-) Nicotine**	341 ± 69 (n = 2)	526 ± 58 (n = 3)

### Selectivity Profile at 5-HT_3A_ Receptors and Other Targets

Potent displacement of specific [^3^H]-granisetron binding (mean K_i_ ± SD) was observed following incubation with granisetron (1.7 ± 0.5 nM; n = 3), MDL-72222 (9.4 ± 4.9 nM; n = 5) and in preliminary studies examining EVP-6124 (2.5 nM; n = 1; S3 Fig). In contrast BMS-933043 showed much weaker binding affinity at human 5-HT_3A_ receptors (K_i_ = 2,451 ± 197 nM; n = 2; S3 Fig) and TC-5619 was not effective (K_i_ >10 μM). Functional FLIPR assays confirmed that BMS-933043 is a 5-HT_3A_ antagonist with 345 fold functional selectivity for α7 nAChR agonism (Table 1). Consistent with binding data, EVP-6124 potently inhibited 5-HT_3A_ receptor function whereas TC-5619 was not effective (Table 1). BMS-933043 was also examined in a broad range of cell based assays at 37 additional receptor or enzyme targets with no additional pharmacological activity of relevance identified (S2 Table).

### Partial Agonist Profile in Whole Cell Voltage Clamp

**Fig 2:**
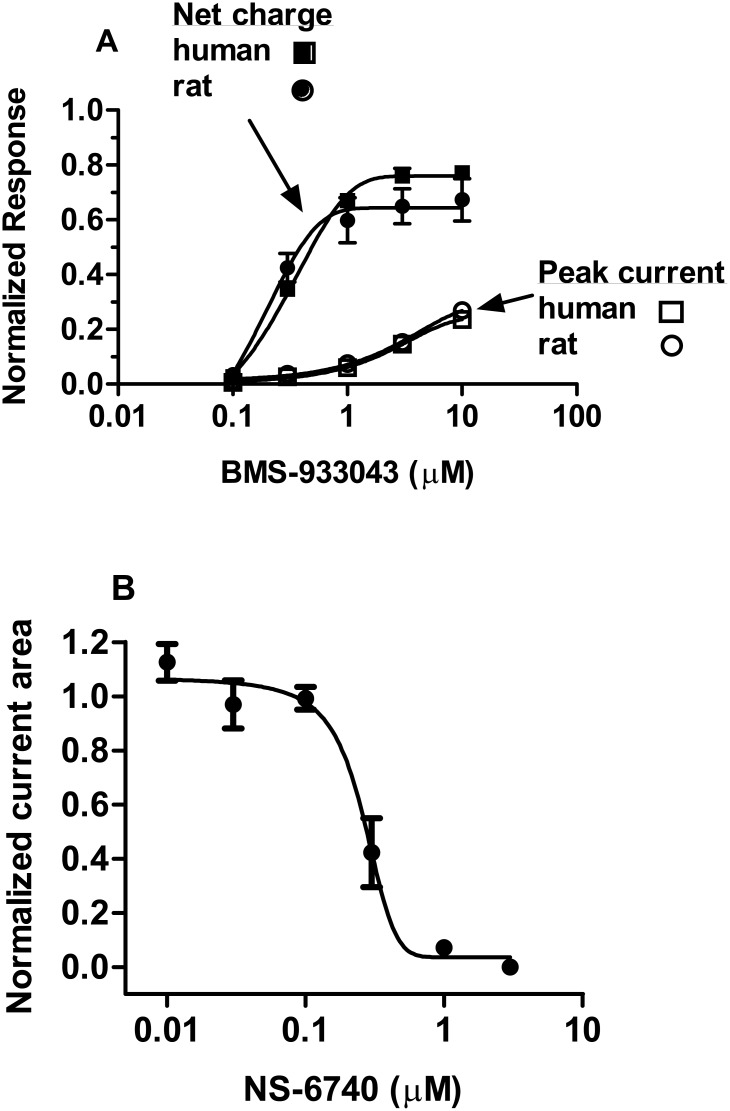
Partial agonist profile of BMS-933043 and inhibition by NS-6740 in whole cell voltage clamp electrophysiology. A) Results show the mean ± SEM peak current response (open symbols) or net charge crossing the membrane (closed symbols) at rat (circles; n = 4) and human (squares; n = 7) α7 nAChR. B) Results show the mean ± SEM net charge crossing the membrane following BMS-933043 (10 μM) application in the presence of increasing concentrations of NS-6740 (n = 6). All results were normalized to the maximal response elicited by ACh.

BMS-933043 exhibited a partial agonist profile when briefly applied to HEK293 cells expressing the human or rat α7 nAChR (Fig 2A). The EC_50_ values determined from the analysis of net charge crossing the cell membrane were 0.29 μM and 0.14 μM respectively (Table 3). BMS-933043 (10 μM) elicited currents in HEK293/rat α7 nAChR cells were inhibited >90% following pre-incubation with 10 nM MLA; the mean ± SEM (n = 5) peak current Ymax was 0.26 ± 0.03 versus 0.02 ± 0.003 in the presence of MLA. BMS-933043 (10 μM) currents were also inhibited following pre-incubation with NS-6740; the NS-6740 IC_50_ values for inhibition of the peak current response and net charge crossing the membrane were 283 ± 33 nM and 255 nM ± 38 (n = 6) respectively (Fig 2B). Activation of α7 nAChR currents was also observed after brief application of EVP-6124 or TC-5619 (S4 Fig) with both agents showing higher levels of intrinsic efficacy compared to BMS-933043 (Table 3).

**Table 3:** Agonist efficacy of BMS-933043, EVP-6124 and TC-5619 determined by patch clamp electrophysiology at recombinant human and rat α7 nAChRs expressed in HEK293 cells.

Agent	Species	Mean ± SEM Peak Current Ymax_obs_ @ 10 μM	Mean ± SEM Net Charge Ymax	Mean ± SEM Net Charge EC_50_ (μM)
**BMS-933043**	Human (n = 7)	0.24 ± 0.03	0.78 ± 0.004	0.29 ± 0.01
	Rat (n = 4)	0.27 ± 0.02	0.67 ± 0.07	0.14 ± 0.03
**EVP-6124**	Human (n = 4)	0.32 ± 0.05	1.36 ± 0.09	0.07 ± 0.02
**TC-5619**	Rat (n = 3)	0.55 ± 0.06	1.26 ± 0.02	0.08 ± 0.01

### Improved Novel Object Recognition Memory in Mice

**Fig 3:**
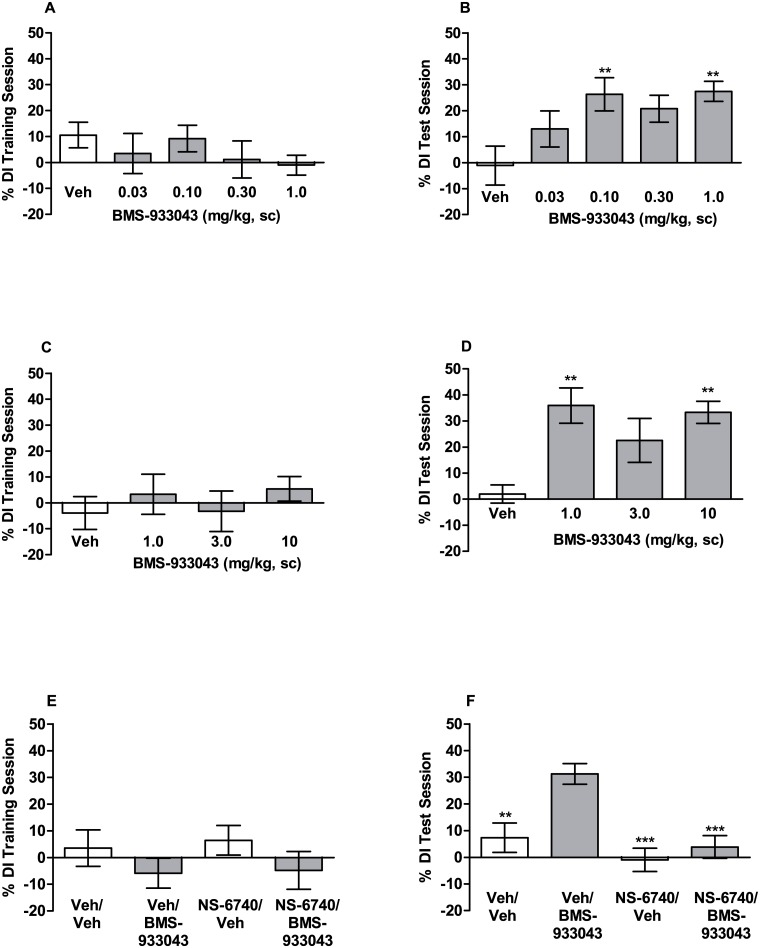
BMS-933043 improves 24 h recognition memory in mice. Subjects were treated 30 min prior to the training session with either A/B) vehicle or low doses of BMS-933043 (n = 11–13/group), C/D) vehicle or high doses of BMS-933043 (n = 10–13/group) or E/F) vehicle or NS-6740 (10 mg/kg, sc) 10 min prior to BMS-933043 (0.3 mg/kg, sc; n = 15–16/group) and then tested for memory retention 24 h later. Results are presented as the mean ± SEM % discrimination index for the training (A, C, E) and test sessions (B, D, F) and were analyzed by ANOVA followed by Dunnett’s test; ** p<0.01, *** p<0.001 versus vehicle or vehicle/BMS-933043 treated mice.

In the novel object recognition procedure mice were treated with either vehicle or BMS-933043 prior to the training session and allowed to explore 2 identical objects. Results for the training session show that the discrimination index was not significantly different for any treatment group (Fig 3A (F(4,56) = 0.73, p = 0.57; Fig 3C (F(3,44) = 0.46, p = 0.71; Fig 3E (F(3,57) = 0.92, p = 0.44). Additional analysis also showed that i) the mean discrimination index did not differ significantly from zero for any treatment group and ii) total object exploration time was similar for all treatment groups (S5 Fig). These results confirm that drug treatment did not affect object exploration time and that mice showed no exploration bias towards any object or its location. Assessment of object recognition memory 24 h after training showed that vehicle treated mice had poor memory retention of the object previously encountered during the training session (Fig 3). In contrast, BMS-933043-treated subjects showed improved memory retention as indicated by a significant increase in the discrimination index. Analysis of variance showed that treatment with BMS-933043, at the dose range of 0.03–1 mg/kg, significantly increased novel object exploration (F(4,56) = 3.65; p = 0.013). Further post-hoc analysis showed a significant increase at the 0.1 mg/kg and 1 mg/kg doses (Fig 3A). Analysis of variance also showed a significant treatment effect at the higher dose range of 1–10 mg/kg (F(3,44) = 5.6; p = 0.0024) which was apparent at doses of 1 mg/kg and 10 mg/kg (Fig 3B). The average total plasma BMS-933043 concentration across the effective dose range tested (0.1–10 mg/kg, sc) was 52 nM—5,555 nM and the compound showed modest brain penetration in mice (average brain/plasma = 17%; S3 Table). Finally, consistent with a silent agonist profile, mice treated with NS-6740 at 3 or 10 mg/kg sc did not show improved 24 h recognition memory (S6 Fig). Independent ex vivo occupancy studies confirmed high levels of α7 nAChR occupancy at these doses (89% and 98% respectively; S6 Fig) consistent with the excellent CNS penetration shown by this agent (average brain/plasma ratio >8; S4 Table). Pretreatment with NS-6740 (10 mg/kg, sc) abolished the improved discrimination index seen in vehicle/BMS-933043 (0.3 mg/kg, sc) treated mice (F(3,57) = 9.6, p <0.0001; Fig 3C). On the basis of these results BMS-933043 was progressed to further evaluation in rodent NMDA receptor deficit models of cognition and sensory processing.

### Reversal of MK-801 Impairment of Set Shifting in a Maze Based Task in Rats

**Fig 4:**
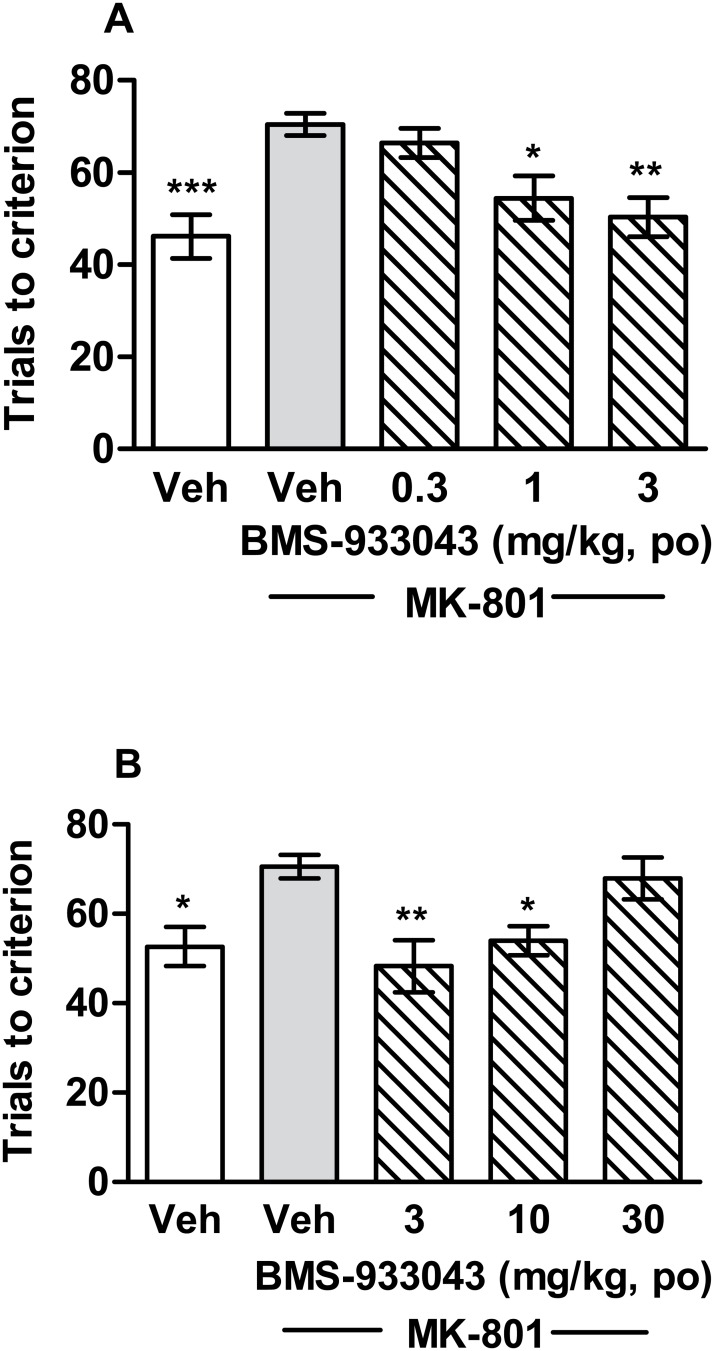
BMS-933043 reverses MK-801-induced impairment of set shifting in rats. A) Rats were treated with vehicle or 0.3–3 mg/kg BMS-933043 or B) vehicle or 3–30 mg/kg BMS-933043 prior to MK-801 administration. Results are expressed as the mean ± SEM number of trials required to reach the performance criterion of 8 successive correct entries into the baited arm (n = 9–10/treatment). Data were analyzed by ANOVA followed by Dunnett’s post hoc analysis; * p<0.05, ** p<0.01, *** p<0.001 versus vehicle/MK-801 treated rats.

All rats were able to achieve performance criteria of 8 successive correct responses on set 1 with no difference in the number of trials required across the groups. On day 2, treatment with MK-801 significantly increased the number of trials required to form the new association between stimulus dimension and reward compared to vehicle-treated rats (Fig 4). Impaired performance accuracy was also associated with a significant increase in perseverative errors (S7 Fig). Oral administration of BMS-933043 significantly improved performance accuracy (Fig 4; low dose study: F(4,44) = 6.85, p = 0.0002; high dose study: F(4,44) = 5.37, p = 0.0013) and decreased perseverative errors in MK-801-treated animals (S7 Fig). BMS-933043 exhibited a U shaped dose response, the minimum effective dose was 1 mg/kg (Fig 4A) and efficacy was lost at 30 mg/kg (Fig 4B). The average total plasma BMS-933043 concentration across the efficacious dose range (1–10 mg/kg, po) was 50 nM—1,860 nM (S3 Table).

### Reversal of MK-801 Deficits in 2 Trial Y Maze Performance in Mice

**Fig 5:**
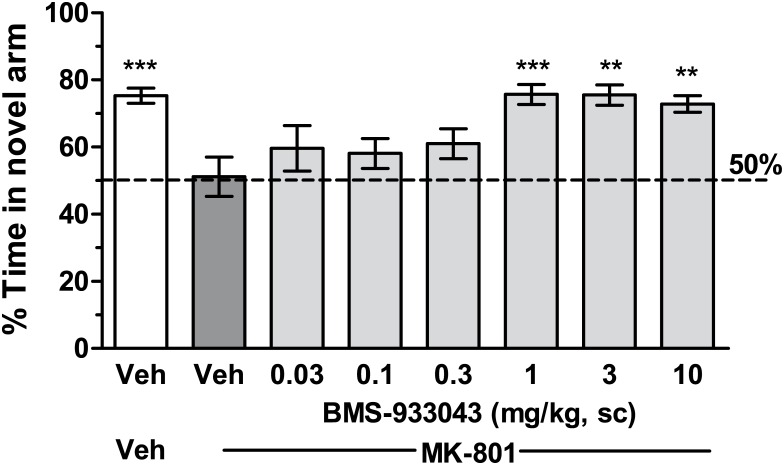
Reversal of MK-801-induced deficits in 2 trial Y maze performance in mice. Results from the free-choice trial are presented as the mean ± SEM % time spent in the novel arm (n = 16–18/treatment) and were analyzed by ANOVA followed by Dunnett’s post hoc analysis; ** p<0.01, *** p<0.001 versus vehicle/MK-801 treatment.

Mice treated with vehicle showed a strong preference for the novel arm spending on average 137 seconds in the novel arm versus 44 seconds in the familiar arm (Fig 5). In contrast, mice treated with MK-801 showed no preference with novel arm exploration at the 50% chance level. Pretreatment with BMS-933043 significantly increased time spent in the novel arm in MK-801-treated mice with efficacy observed at doses of 1, 3 and 10 mg/kg (F(7,128) = 5.09, p<0.0001; Fig 5). The average total plasma BMS-933043 concentration at the efficacious dose range was 254 nM—2,982 nM (S3 Table).

### Reversal of S(+)Ketamine Deficits in N40 Gating in Rats

**Fig 6:**
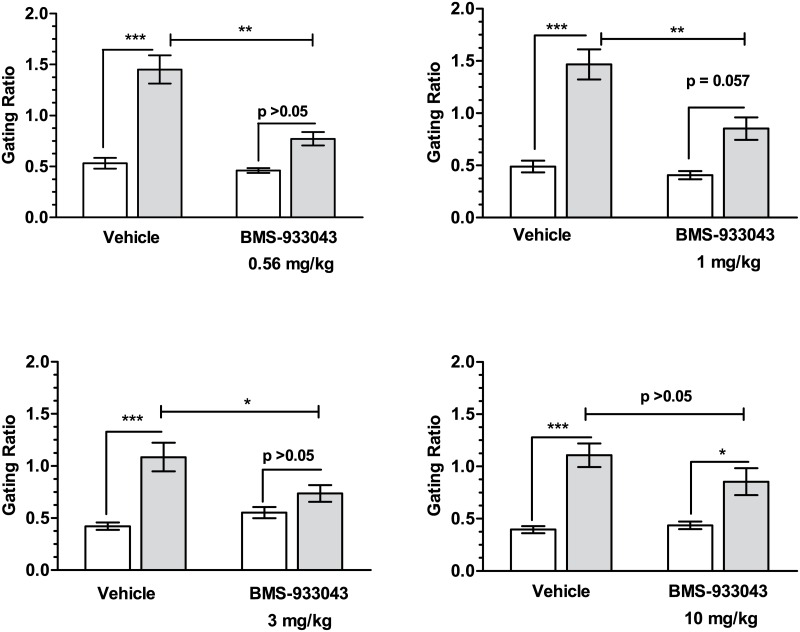
Reversal of S(+)ketamine deficits in N40 gating in rats. Results for 4 independent studies (n = 12–13/study) are presented as the mean ± SEM gating ratio (S2/S1) determined after pretreatment with vehicle or BMS-933043 prior to (ie baseline; white bars) or following subsequent S(+)ketamine administration (black bars). Data were analyzed by 2 way repeated measures ANOVA followed by Sidak’s multiple comparison test; * p<0.05, ** p<0.01, *** p<0.001.

Baseline gating was not different under BMS-933043 or vehicle treatment conditions (Fig 6). In contrast, administration of S(+)ketamine (10 mg/kg, sc) produced a robust disruption in auditory gating in vehicle-pretreated rats as shown by a significant increase in the S2/S1 ratio compared to the pre-ketamine response. Pretreatment with BMS-933043 attenuated the deficit in N40 gating following S(+)ketamine treatment with a significant reduction seen at 0.56 mg/kg (treatment effect: F(1,12) = 20.12, p = 0.0007; ketamine effect: F(1,12) = 76.51, p<0.0001; treatment x ketamine interaction: F(1,12) = 10.19, p = 0.0077), 1 mg/kg (treatment effect: F(1,12) = 18.42, p = 0.001; ketamine effect: F(1,12) = 53.37, p<0.0001; treatment x ketamine interaction: F(1,12) = 6.722, p = 0.0235) and 3 mg/kg (treatment effect: F(1,12) = 2.612, p = 0.132; ketamine effect: F(1,12) = 20.57, p = 0.0007; treatment x ketamine interaction: F(1,12) = 9.425, p = 0.0097). In contrast neither the lowest dose (0.3 mg/kg; data not shown) nor the highest dose (10 mg/kg) tested were significantly different indicating a U shaped dose-effect relationship in this model. The average total plasma BMS-933043 concentration at the efficacious dose range (0.56–3 mg/kg, sc) was 146 nM—1,360 nM and BMS-933043 showed modest CNS penetration in rats (average brain/plasma for all rat studies = 28%, S3 Table).

### Restoration of ID/ED Set Shifting in Neonatal PCP-Treated Rats

**Fig 7:**
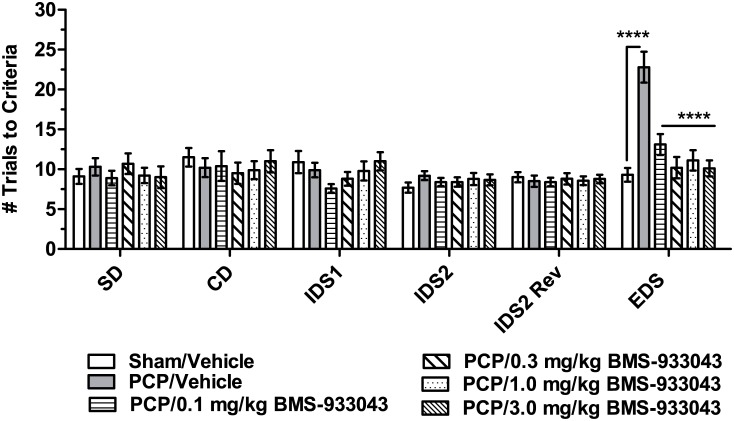
BMS-933043 improves EDS performance in neonatal PCP-treated rats. Results are presented as the mean ± SEM number of trials required to reach the performance criterion of 6 successive correct entries into the baited pot for each discrimination (n = 9–10/treatment). Results were analyzed by 2 way repeated measures ANOVA followed by Dunnett’s post hoc analysis; **** p = 0.0001 compared to neonatal PCP/Vehicle treated rats.

Analysis of trials to criteria by 2 way repeated measures ANOVA showed a significant effect of treatment (F(5,52) = 3.61, p = 0.007), discrimination (F(5,260) = 13.67, p<0.0001) and a significant treatment x discrimination interaction (F(25,260) = 4.59, P<0.0001). Dunnett’s post-hoc analysis showed that neonatal PCP-treated rats were selectively impaired at the EDS stage but otherwise performed all discriminations similarly to sham/vehicle treated subjects (Fig 7). Treatment with BMS-933043 significantly improved EDS performance in neonatal PCP treated rats. The impairment at the EDS stage and the improvement following BMS-933043 treatment was similar for rats shifting from odor to media or visa versa (S8 Fig). Neonatal PCP treated rats also took significantly longer to complete the EDS compared to all other discriminations and this effect was also attenuated by BMS-933043 treatment (S9 Fig). The average total plasma concentration across the range of doses of BMS-933043 examined was <3.7 nM—225 nM (S3 Table).

### Improved Deviance Detection in Neonatal PCP Treated Rats

**Fig 8:**
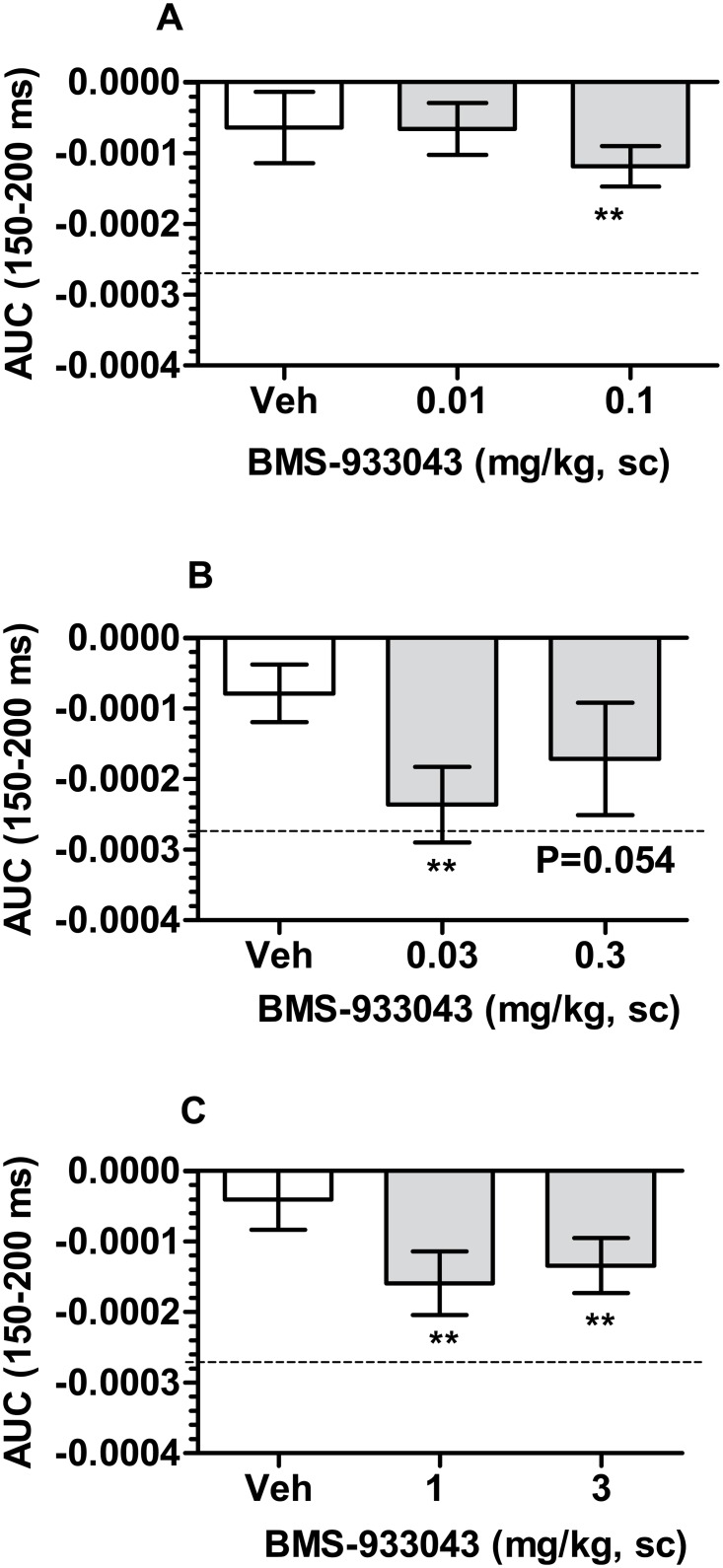
BMS-933043 improves MMN in neonatal PCP-treated rats. Results from 3 independent studies are presented as the mean ± SEM AUC determined from the averaged difference wave for each subject after treatment with either vehicle or BMS-933043 (n = 12–13/group). For reference, the dashed line shows the average AUC in untreated adult rats determined in separate studies. Each treatment was analyzed by one sample, two tailed t test; ** p<0.01 compared to a hypothetical zero.

Rats treated neonatally with PCP showed negligible deviance detection after vehicle administration (Fig 8). While not examined in the present studies, we have previously demonstrated robust deviance detection in adult Sprague Dawley rats [40]. Following subcutaneous administration of BMS-933043, significant deviance detection from a hypothetical zero was observed at doses of 0.03, 0.1, 1 and 3 mg/kg with the effect of 0.3 mg/kg just failing to achieve statistical significance (Fig 8). The average total plasma BMS-933043 concentration across the efficacious dose range (0.03–3 mg/kg, sc) was 23 nM—1,974 nM (S3 Table).

### Ex Vivo Occupancy/Exposure Relationships in Rodents

**Fig 9:**
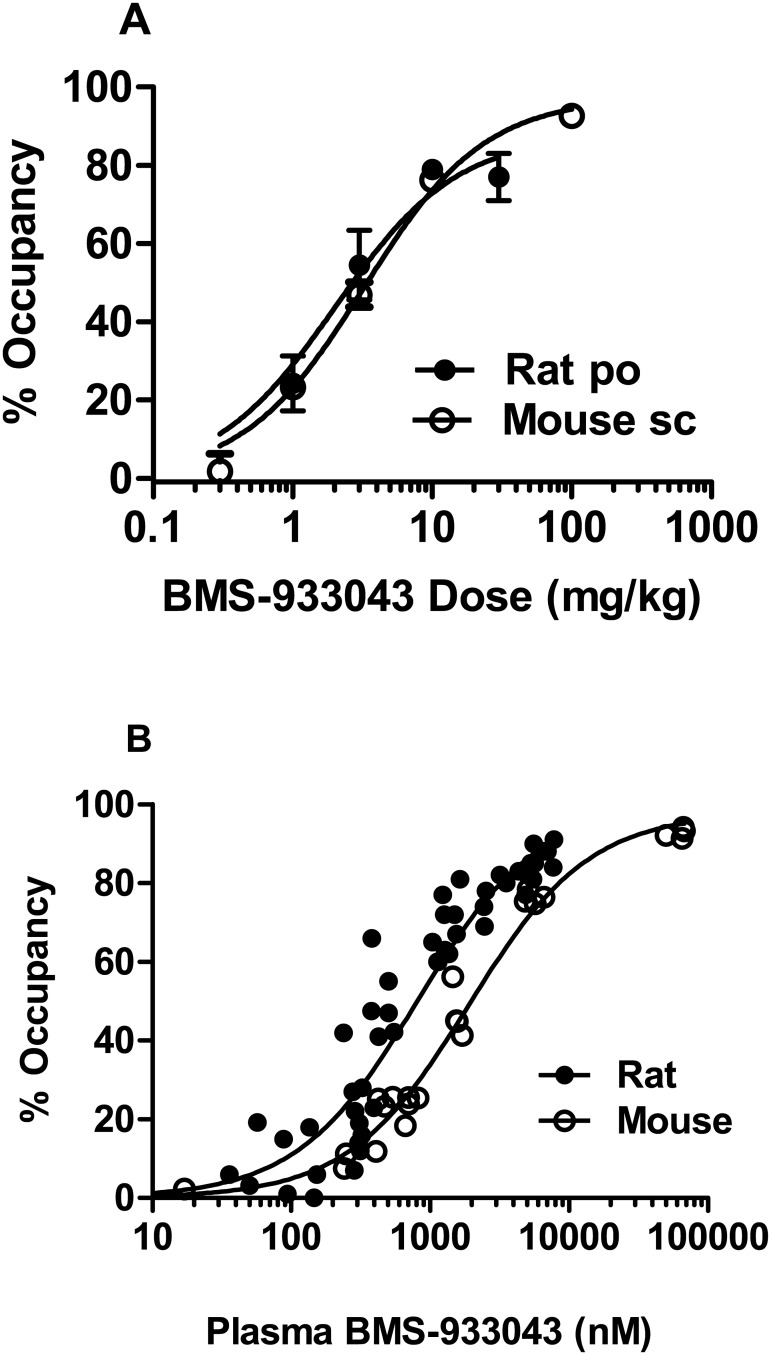
Relationship between dose, plasma exposure and ex vivo α7 nAChR occupancy in rodents. A) Results show the mean ± SEM occupancy determined 30 min after sc dosing in mice (open circles) or 90 min after po dosing in rats (n = 4/dose). B) Individual occupancy/exposure results for mice (open circles) and composite plot showing results for rats (closed circles) treated po (as above), sc (30 min post-dose) and experimental set shift subjects (150 min post-dose).

[^3^H]-A-585539 binding can be conducted on brain tissue collected from rodents dosed with α7 nACh receptor agents to provide a measure of *ex vivo* receptor occupancy. Under the assay conditions, the specific/non-specific binding window determined in forebrain homogenates from vehicle-treated rodents was >5–6 fold and time course studies showed a consistent level of occupancy over a 20 min incubation period suggesting minimal dissociation of test agent. BMS-933043 treatment inhibited *ex vivo* [^3^H]A-585539 binding in a dose-dependent manner after po dosing in rats or sc dosing in mice (Fig 9A). In both cases, occupancy was first detected (~20%) at 1 mg/kg with >75% occupancy achieved at 10 mg/kg. Occupancy was strongly correlated with plasma exposure; the total plasma concentration achieving 50% *ex vivo* α7 nAChR occupancy was 810 nM and 1.9 μM in rats and mice respectively (Fig 9B). The plasma exposures associated with improved cognition and sensory processing are summarized in Table 4 and were determined in experimental subjects or satellite groups dosed in parallel (n = 4/dose).

**Table 4:** Summary of BMS-933043 Results in Preclinical Models of Schizophrenia.

Model	Impairment Approach	Active dose range (mg/kg)	Average plasma concentration range (subjects; time post-dose)
**MK-801 set shifting**	Acute NMDA deficit	1–10 (po)	50–1,860 nM (experimental; 150 min)
**MK-801 Y maze**	Acute NMDA deficit	1–10 (sc)	254–2,982 nM (experimental; 60 min)
**S(+)Ketamine N40 gating**	Acute NMDA deficit	0.56–3.0 (sc)	146–1,360 nM (satellite; 30 min)
**Neonatal PCP ID/ED**	Developmental	0.1–3.0 (po)	<3.07–225 nM (experimental; 150 min)
**Neonatal PCP MMN**	Developmental	0.03–3.0 (sc)	23–1,974 nM (satellite; 30 min)

## Discussion

The development of α7 nAChR agonists is widely recognized as a promising approach for the treatment of cognitive impairment in schizophrenia patients. In the present studies we present the preclinical profile of BMS-933043, a novel, highly selective α7 nAChR partial agonist.

To characterize the *in vitro* profile of BMS-933043 we determined binding affinity, functional potency, intrinsic efficacy and receptor selectivity. Radioligand binding studies were conducted using the α7 nAChR agonist radioligand, [^3^H]-A-585539, and showed that BMS-933043 has high affinity for rat (3 nM) and human (8 nM) α7 nAChRs. Consistent with published results, [^3^H]-A-585539 labeled a single site with high affinity; in rat brain the Kd was similar to previous reports although the Kd at recombinant human α7 nAChRs was about 10 fold lower than human cortex [33]. Specific binding of [^3^A]-585539 was fully displaced by several α7 nAChR agents with Ki values at rat α7 nAChRs consistent with previous results using this agonist radioligand [33, 41]. Furthermore, the higher affinities seen in the present studies for TC-5619 (reported Ki = 1 nM; [23]) and EVP-6124 (reported Ki = 10 nM; [24]) are consistent with the 5–10 fold higher affinity observed when using this radioligand versus [^3^H]MLA [33].

In functional studies BMS-933043 showed an agonist profile and produced a concentration dependent increase in Ca^2+^ flux in FLIPR assays conducted on HEK293/rat α7 nAChR cells. Agonist-induced responses were also observed after application of several other agents with a rank order of potency similar to that observed in binding assays. Further characterization using voltage clamp electrophysiology showed that BMS-933043 exhibits a partial α7 nAChR agonist profile with a maximal increase in net charge 67–78% of the ACh response. In comparison, EVP-6124 and TC-5619 showed higher intrinsic efficacy; results which appear consistent with studies conducted in oocytes expressing recombinant human α7 nAChRs [23–24]. In addition, while the primary measure (i.e. peak versus net charge) varies, the EC_50_ values determined in the present study are consistent with published results and show a shift relative to α7 nAChR binding affinity that is usually observed for these agents. Interestingly we also noted that a 1 min wash was sufficient for α7 nAChR currents to fully recover from desensitization after BMS-933043 application which was not the case for EVP-6124 or TC-5619. Further detailed investigation of the functional and binding kinetics for these agents will be important to establish whether recovery from desensitization is significantly faster for BMS-933043.

Evaluation at other nAChR subtypes and a panel of 37 additional receptor or enzyme targets confirmed that BMS-933043 is a selective α7 nAChR agonist. Furthermore BMS-933043 retained a >300 fold selectivity window over the closely related 5-HT_3A_ receptor both with respect to binding affinity and functional antagonism. Drugs that antagonize 5-HT_3_ receptors are known to produce constipation which was also reported in patients with Alzheimer’s Disease treated with RG3487 and EVP-6124 [42]. Since both agents potently bind to and inhibit 5-HT_3A_ receptors in vitro it seems likely that this mechanism mediates the gastrointestinal side effects observed. Whether this liability ultimately leads to suboptimal therapeutic dosing remains to be seen but the improved selectivity window suggests that BMS-933043 is unlikely to produce constipation in humans.

To examine pro-cognitive effects, BMS-933043 was first evaluated in NOR, a model of object recognition memory in mice. In these studies BMS-933043 was administered prior to training only and memory retention evaluated 24 hours later. While the results show that BMS-933043 can improve memory acquisition/consolidation under these treatment conditions, the dose response relationship was inconsistent with some intermittent doses failing to achieve statistical analysis. This profile may result from the relatively high variability in the data since less rigorous statistical analysis showed a significant increase in novel versus familiar object exploration time and a significant increase in % DI from hypothetical zero for these doses. In addition, BMS-933043 was robustly efficacious in NMDA receptor mediated deficit models suggesting that enhancement of memory retention in normal, non-impaired subjects may be more challenging to demonstrate. Additional studies, with increased number of subjects may be required to more fully define the NOR dose response relationship however the improvement observed was reproducible and sufficient to explore the mechanism of action of BMS-933043 using NS-6740. NS-6740 has been described as a silent agonist; in functional assays it shows minimal agonist activity *per se*, but elicits channel activation when applied under conditions that de-stabilize channel desensitization e.g. co-application with the positive allosteric modulator, PNU-120596, or application to the slowly desensitizing mutant receptor α7V274T [41, 43]. Thus while NS-6740 binds to the orthosteric binding site, this agent preferentially drives the channel to a non-conducting desensitized state, a profile that has enabled it to be used in vivo to effectively block α7 nAChR agonist responses [41]. In the present studies we also show that NS-6740 has high binding affinity for the α7 nAChR orthosteric site, does not activate Ca^2+^ flux in HEK293/α7 nAChR cells, inhibits BMS-933043 elicited currents and achieves high levels of α7 nAChR occupancy consistent with its excellent brain penetration (brain/plasma ratio >8). The CNS penetration of NS-6740 is superior to MLA which shows poor CNS penetration in rodents (brain/plasma = 0.06–0.09) [44] and only modest α7 nAChR occupancy in our *ex vivo* studies after dosing in mice (16% at 10 mg/kg; unpublished results). Finally, consistent with previous results [41] we also show that NS-6740 can be used as an in vivo tool to block α7 nAChR agonist effects since this agent reversed the improvement in recognition memory following BMS-933043 treatment. These results are thus consistent with α7 nAChR channel activation as the primary mechanism driving the pro-cognitive effect of BMS-933043 in the mouse NOR paradigm.

We then proceeded to evaluate BMS-933043 in acute, NMDA antagonist induced deficit models of executive function, working memory and sensory gating and showed a robust improvement in all cases. Firstly BMS-933043 treatment fully reversed the impairment in attentional set shifting induced by MK-801 in a maze-based task requiring animals to modify behavior based on either the color or texture of individual arms. This procedure is similar to the Wisconsin Card Sort Task used in humans to examine attentional set shifting and also depends critically on the prefrontal cortex [36]. The robust improvement seen with BMS-933043 treatment is consistent with previous studies from our laboratory showing that the α7 nAChR agonists SSR-180711, PNU-282987 and GTS-21 are also effective in this model [19]. To examine effects on working memory we used a continuous Y maze task, a model previously used to demonstrate reversal of scopolamine-induced performance deficits following α7 nAChR agonist treatment [39]. In contrast to the NOR procedure, the Y maze task has minimal delay between the forced choice training trial and the free choice test trial and is considered an attentional/working memory task [39]. Using this paradigm we showed that a low dose of MK-801 also reduced the time spent in the novel arm during the free choice trial indicative of a working memory impairment, an effect which was reversed by BMS-933043 treatment. Finally we showed that BMS-933043 alleviates the N40 gating deficit induced by S(+)ketamine in rats, an effect consistent with a wide body of literature demonstrating that α7 nAChR agonists improve sensory processing in pharmacological deficit models and genetic models such as DBA/2 mice [45].

In a final set of studies we examined the effects of BMS-933043 in a neurodevelopmental model of schizophrenia involving neonatal treatment with PCP. This procedure has been shown to result in a number of behavioral changes including cognitive impairment in attentional set shifting paradigms such as the ID/ED test. Like the maze-based task, the ability to shift behavior between attentional sets in the ID/ED procedure is critically dependent on the mPFC [35]. Furthermore, neonatal PCP treated rats are selectively impaired at the EDS, but otherwise perform all other discriminations in the ID/ED task at the same level as control subjects [37–38]. Similar to the findings in schizophrenia patients, these animals also show a reduction in mPFC levels of the GABA marker parvalbumin and impaired GABAergic inhibition of mPFC pyramidal neurons which is thought to underlie the selective EDS impairment seen in these subjects [46–47]. In the present study we also show a selective EDS impairment in neonatal PCP treated rats that was completely reversed by all doses of BMS-933043. The lack of dose responsiveness compared to other models was unexpected but similar to the ID/ED results reported for RG-3487 in rats treated subchronically with PCP suggesting high sensitivity to these agents [18]. It is also important to note that a worsening of performance between the IDS2 and EDS in sham controls was not observed in our study suggesting that the development of an attentional set was not achieved. Therefore additional studies are needed to clarify the effectiveness of BMS-933043 in this model. We also examined MMN in neonatal PCP treated rats using a paradigm previously reported by our laboratory to produce robust MMN in normal Sprague Dawley rats [40]. Our results show that MMN was absent in neonatal PCP subjects but was restored by treatment with BMS-933043. To our knowledge this is the first demonstration that neonatal PCP treated rats have reduced MMN, a deficit which is also seen in schizophrenia patients. Furthermore, since MMN deficits can be improved in patients treated with the α7 nAChR agonist EVP-6124, our results suggest that this preclinical model may serve as a translational approach to explore MMN as a translational pharmacodynamic biomarker for this mechanism [31].

While BMS-933043 was effective in the preclinical models examined, several observations highlight the challenges associated with using preclinical data to predict clinically efficacious doses for this mechanism. Firstly, the minimal effective dose varied with the neonatal PCP neuro-developmental model being the most sensitive to BMS-933043 treatment. In contrast, reversal of acute NMDA antagonist-induced impairment generally required higher doses for both gating and cognition measures. Secondly, while not fully examined in all studies, a U shaped dose-response was observed in acute NMDA antagonist-treated rats. The potential for U shaped dose-response curves is well recognized for this mechanism and is thought to reflect high levels of target occupancy/activation leading to persistent receptor desensitization. Indeed, at doses associated with a loss of efficacy, average plasma concentrations ranged from 3,307 nM (10 mg/kg, sc; N40 gating) to 7,255 nM (30 mg/kg, po; set shift) corresponding to *ex vivo* α7 nAChR occupancy ≥80% in rats. Across the models the plasma concentration range over which BMS-933043 retained efficacy varied; in acute NMDA antagonist models the exposure range was 10–37 fold while in the remaining models the range was >70 fold suggesting that a broad dose range should be examined in humans to define this relationship. Finally, while a clear relationship between brain α7 nAChR *ex vivo* occupancy and plasma exposure was observed in rodents, preclinical efficacy at the minimum effective dose was associated with very low levels of target engagement. While similar results have also been reported for A-582941 using *ex vivo* [^3^H]BTX binding [39] other α7 nAChR agonists appear to require higher occupancy for efficacy using this approach [39, 48]. These results suggest that the occupancy/efficacy relationship may be compound specific and further work is needed to understand whether this reflects biological differences or the potential limitations of *ex vivo* radioligand binding techniques. Further, while new PET radiotracers such as [^18^F]ASEM have been reported [49] the present results highlight the important role of translational pharmacodynamic biomarkers such as MMN and sensory gating and the potential limitations of PET imaging studies for agents like BMS-933043.

In conclusion, BMS-933043 is a novel, potent and selective α7 nAChR partial agonist that reliably improves cognition and sensory processing in preclinical models of schizophrenia. On the basis of these results, together with a favorable preclinical safety profile, BMS-933043 was selected for further development and has progressed to clinical evaluation in humans.

## Supporting Information

S1 Dataset Individual human α7 nAChR EC_50_ or 5-HT_3A_ IC_50_ values determined in Ca^2+^ flux (FLIPR) assays. (PDF)

S2 Dataset Individual Ki values determined by radioligand binding at rat or human α7 nAChR and human 5-HT_3A_ receptors. (PDF)

S3 Dataset Individual peak current and current area (net charge) values in HEK293 cells expressing human or rat α7 nAChR determined by patch clamp electrophysiology. (PDF)

S4 Dataset % Discrimination Index (DI) results for individual subjects evaluated in mouse NOR after BMS-933043 treatment (0.03–1 mg/kg). (PDF)

S5 Dataset % Discrimination Index (DI) results for individual subjects evaluated in mouse NOR after BMS-933043 treatment (1–10 mg/kg). (PDF)

S6 Dataset % Discrimination Index (DI) results for individual subjects evaluated in mouse NOR after treatment with NS-6740 and BMS-933043. (PDF)

S7 Dataset Number of trials to criteria for individual subjects evaluated in the MK-801 set shift model after treatment with BMS-933043. (PDF)

S8 Dataset % Time spent in the novel arm for individual mice evaluated in the MK-801 Y maze procedure after treatment with BMS-933043. (PDF)

S9 Dataset Gating ratio for individual subjects evaluated in the S(+)ketamine N40 gating model after treatment with BMS-933043. (PDF)

S10 Dataset Number of trials to criteria at each discrimination stage for individual rats evaluated in the neonatal PCP ID/ED model. (PDF)

S11 Dataset Individual AUC (150–200 ms) results for neonatal PCP treated rats evaluated in the MMN model after treatment with BMS-933043. (PDF)

S12 Dataset Ex vivo α7 nAChR receptor occupancy results for individual subjects. (PDF)

S1 Fig Representative mean evoked response potential waveforms and calculation of the AUC to determine treatment effects on MMN in neonatal PCP treated rats. (PDF)

S2 Fig Inhibition of [^3^H]A-585539 binding to α7 nACh receptors. (PDF)

S3 Fig Inhibition of [^3^H]granisetron binding to human 5-HT_3A_ receptors. (PDF)

S4 Fig Agonist profile of EVP-6124 and TC-5619 in voltage clamp electrophysiology. (PDF)

S5 Fig Treatment with BMS-933043 does not alter total object exploration time in the training session or testing session in mice. (PDF)

S6 Fig NS-6740 does not impact NOR performance at doses achieving high levels of α7 nAChR occupancy in mice. (PDF)

S7 Fig BMS-933043 reduces perseverative errors in MK-801 treated rats performing the maze based set shifting task. (PDF)

S8 Fig EDS impairment and improvement following BMS-933043 treatment is similar in neonatal PCP treated rats shifted from media to odor or odor to media at the EDS stage. (PDF)

S9 Fig BMS-933043 decreases the time to complete the EDS discrimination in neonatal PCP treated rats. (PDF)

S1 Table Example of exemplars used at each discrimination phase of the rat ID/ED task. (PDF)

S2 Table Pharmacological evaluation of BMS-933043 at other receptor and enzyme targets. (PDF)

S3 Table Plasma and brain concentrations of BMS-933043 determined after subcutaneous or oral dosing in rodents. (PDF)

S4 Table Plasma and brain concentrations of NS-6740 determined after subcutaneous dosing in mice. (PDF)
