# Supplementary material for: The Novel, Nicotinic Alpha7 Receptor Partial Agonist, BMS-933043, Improves Cognition and Sensory Processing in Preclinical Models of Schizophrenia
Source: PLoS One. 2016 Jul 28;11(7):e0159996. doi: 10.1371/journal.pone.0159996 (PMC4965148; doi:10.1371/journal.pone.0159996)
Supplement: S1 Dataset — (PDF) [file pone.0159996.s001.pdf]

S1 Dataset. Individual human α7 nAChR EC_{50} or 5-HT_{3A} IC_{50} values determined in Ca^{2+} flux (FLIPR) assays.

Compound	n	Human α7 nAChR EC_{50} values (nM)
BMS-933043	16	13.4; 21.4; 9.4; 30.5; 15.1; 50.0; 19.2; 14.8; 18.2; 17.3; 28.2; 17.0; 23.3; 28.7; 40.8; 27.3
EVP-6124	4	16.3; 29.4; 4.0; 9.0
TC-5619	5	1.9; 5.0; 6.6; 2.5; 3.3
A-582941	6	92.1; 189.2; 101.3; 69.3; 41.0; 45.8
PNU-282987	9	105.8; 98.2; 88.4; 74.4; 81.4; 117.8; 46.0; 145.4; 92.8
NS-6740	7	>100,000; >100,000; >100,000; >100,000; >100,000; >100,000
**Compound**	**n**	**Human 5-HT_{3A} IC_{50} values (µM)**
BMS-933043	10	10.54; 7.20; 8.02; 6.88; 7.28; 4.89; 12.44; 5.32; 8.67; 9.42
EVP-6124	5	0.006; 0.003; 0.001; 0.004; 0.005
TC-5619	9	>100; >100; >100; >100; >100; >100; >100; >100; >100
A-582941	10	0.086; 0.344; 0.072; 0.073; 0.075; 0.076; 0.109; 0.047; 0.079; 0.059
PNU-282987	5	32.065; 23.639; 6.347; 38.428; 24.115
NS-6740	9	4.699; 5.682; 4.425; 6.208; 5.694; 5.320; 5.908; 2.390; 3.114
