# Supplementary material for: The Novel, Nicotinic Alpha7 Receptor Partial Agonist, BMS-933043, Improves Cognition and Sensory Processing in Preclinical Models of Schizophrenia
Source: PLoS One. 2016 Jul 28;11(7):e0159996. doi: 10.1371/journal.pone.0159996 (PMC4965148; doi:10.1371/journal.pone.0159996)
Supplement: S2 Dataset — (PDF) [file pone.0159996.s002.pdf]

S2 Dataset. Individual Ki values determined by radioligand binding at rat or human α7 nAChR and human 5-HT$_{3A}$ receptors.

Compound	n	Rat Brain α7 nAChR Ki (nM)
BMS-933043	6	2.67; 2.99; 3.08; 3.74; 3.20; 3.82
Epibatidine	6	3.90; 5.40; 5.0; 6.10; 5.90; 5.10
TC-5619	2	0.182; 0.279
EVP-6124	2	0.529; 1.00
A-582941	2	23; 20.3
MLA	2	0.30; 0.20
NS-6740	3	2.45; 2.78; 3.33
(-) Nicotine	2	389; 292
Compound	n	HEK293/human α7 nAChR Ki (nM)
BMS-933043	4	10.5; 5.7; 8.1; 8.1
Epibatidine	4	12.9; 9.8; 9.7; 9.7
TC-5619	2	1.0; 0.8
EVP-6124	2	2.2; 3.8
A-582941	3	99; 72.5; 76.3
MLA	3	4.3; 4.3; 3.9
(-) Nicotine	3	551; 567; 460
Compound	n	HEK293/human 5-HT$_{3A}$ Ki (nM)
BMS-933043	2	2,312; 2,590
EVP-6124	1	2.5
TC-5619	1	>10,000
Granisetron	3	1.2; 1.7; 2.2
MDL-72222	5	6; 7.3; 6.6; 9.2; 18
