# Supplementary material for: The Novel, Nicotinic Alpha7 Receptor Partial Agonist, BMS-933043, Improves Cognition and Sensory Processing in Preclinical Models of Schizophrenia
Source: PLoS One. 2016 Jul 28;11(7):e0159996. doi: 10.1371/journal.pone.0159996 (PMC4965148; doi:10.1371/journal.pone.0159996)
Supplement: S3 Dataset — (PDF) [file pone.0159996.s003.pdf]

S3 Dataset: Individual peak current and current area (net charge) values in HEK293 cells expressing human or rat α7 nAChR determined by patch clamp electrophysiology.

Rat α7 nAChR/HEK293 cells: BMS-933043 Peak Current normalized to 3 mM ACh				
0.1 µM	0.011578	0.010832	0.012359	0.013095
0.3 µM	0.04784	0.028294	0.032838	0.036686
1 µM	0.08082	0.071105	0.073558	0.074896
3 µM	0.155794	0.151942	0.165138	0.13648
10 µM	0.2731	0.29042	0.295206	0.215272
Rat α7 nAChR/HEK293 cells: BMS-933043 Net Charge (Current Area) normalized to 300 µM ACh				
0.1 µM	0.00864	-0.014	0.059171	0.087701
0.3 µM	0.413801	0.285276	0.473938	0.525558
1 µM	0.717234	0.355507	0.662611	0.654857
3 µM	0.791905	0.479351	0.667657	0.656892
10 µM	0.826588	0.463709	0.732215	0.667285

Human α7 nAChR/HEK293 cells: BMS-933043 Peak Current normalized to 3 mM ACh							
0.1 µM	0.005961	0.006675	0.008382	0.007317	0.006875	0.005721	0.00606
0.3 µM	0.026371	0.023027	0.023888	0.024376	0.029505	0.03259	0.027371
1 µM	0.054785	0.032937	0.043924	0.05131	0.085041	0.080244	0.072721
3 µM	0.126023	0.099704	0.109235	0.099127	0.19658	0.201977	0.18297
10 µM	0.216951	0.153151	0.170151	0.177877	0.333392	0.298713	0.30045
Human α7 nAChR/HEK293 cells: BMS-933043 Net Charge (Current Area) normalized to 300 µM ACh							
0.1 µM	0.019825	0.013184	0.038657	0.037817	0.022826	0.013267	0.038558
0.3 µM	0.349601	0.36236	0.321236	0.321544	0.295406	0.357581	0.414317
1 µM	0.695221	0.630407	0.655398	0.612181	0.619044	0.659231	0.812854
3 µM	0.794676	0.779117	0.765452	0.70252	0.69028	0.699137	0.88799
10 µM	0.80353	0.753716	0.796899	0.763672	0.722352	0.678848	0.882297

Rat α7 nAChR/HEK293 cells: NS-6740 Inhibition of BMS-933043 Peak Current normalized to BMS-933043 10 µM alone						
10 nM	1.119375	1.054216	0.873004	1.024836	1.218392	1.26404
30 nM	1.065267	1.128172	1.034426	1.099443	1.466008	0.918053
100 nM	1.201133	1.193386	1.131201	0.996583	1.193301	1.047291
300 nM	1.080938	0.455813	0.828201	0.196243	0.331568	0.197239
1 µM	0.096116	0.087506	0.101631	0.096465	0.170634	0.061213
3 µM	0.054793	0.084062	0.113831	0.060629	0.090642	0.044333
Rat α7 nAChR/HEK293 cells: NS-6740 Inhibition of BMS-933043 Net Charge (Current Area) normalized to BMS-933043 10 µM alone						
10 nM	0.936133	1.053484	1.037169	1.061256	1.326628	1.336793

30 nM	0.823193	0.964182	0.851603	1.057142	1.360053	0.761557
100 nM	0.872405	0.941266	1.090588	0.886582	1.080852	1.082328
300 nM	0.869644	0.370161	0.746063	0.150527	0.186606	0.211792
1 μM	0.047941	0.064529	0.132148	0.068485	0.059664	0.059869
3 μM	-0.00202	-0.01005	-0.01049	0.007827	0.00378	0.007194

Human α7 nAChR/HEK293 cells: EVP-6124 Peak Current normalized to 3 mM ACh				
0.03 μM	0.023095	0.030321	0.023284	0.013159
0.1 μM	0.048497	0.067042	0.097102	0.041302
0.3 μM	0.071567	0.107206	0.154024	0.076664
1 μM	0.127042	0.144738	0.254666	0.142149
3 μM	0.187737	0.244068	0.383192	0.255804
10 μM	0.243764	0.265356	0.462654	0.303426
Human α7 nAChR/HEK293 cells: EVP-6124 Net Charge (Current Area) normalized to 300 μM ACh				
0.03 μM	0.163264	0.206418	0.115292	0.134195
0.1 μM	0.840485	1.081258	0.847891	0.872342
0.3 μM	1.200793	1.30184	1.444892	1.418354
1 μM	1.413753	1.498727	1.608322	1.442349
3 μM	1.219895	1.197068	1.303621	1.069781
10 μM	0.34903	0.405457	0.393906	0.326985

Rat α7 nAChR/HEK293 cells: TC-5619 Peak Current normalized to 3mM ACh			
0.001 μM	0.003543	0.011618	0.003662
0.003 μM	0.003116	0.008701	0.005453
0.01 μM	0.006073	0.007536	0.009875
0.03 μM	0.020042	0.015667	0.021391
0.1 μM	0.054081	0.04485	0.051832
0.3 μM	0.159873	0.12415	0.114915
1 μM	0.276001	0.257587	0.267948
3 μM	0.4166	0.427866	0.455481
10 μM	0.484663	0.485054	0.673182
Rat α7 nAChR/HEK293 cells: TC-5619 Net Charge (Current Area) normalized to 300 μM ACh			
0.001 μM	0.012908	0.179462	0.008199
0.003 μM	-0.00013	0.032411	0.001885
0.01 μM	0.000016	0.002188	-0.00959
0.03 μM	0.149155	0.14684	0.202789
0.1 μM	0.687896	0.723864	0.848076
0.3 μM	1.092841	1.106901	1.105128
1 μM	1.241725	1.14323	1.297158
3 μM	1.233945	1.270866	1.311737
10 μM	1.226251	1.317075	1.306027
