# Supplementary material for: The Novel, Nicotinic Alpha7 Receptor Partial Agonist, BMS-933043, Improves Cognition and Sensory Processing in Preclinical Models of Schizophrenia
Source: PLoS One. 2016 Jul 28;11(7):e0159996. doi: 10.1371/journal.pone.0159996 (PMC4965148; doi:10.1371/journal.pone.0159996)
Supplement: S4 Dataset — (PDF) [file pone.0159996.s004.pdf]

S4 Dataset. % Discrimination Index (DI) results for individual subjects evaluated in mouse NOR after BMS-933043 treatment (0.03 - 1 mg/kg).

Session	% Discrimination Index (DI)				
	Vehicle	0.03 mg/kg BMS-933043	0.1 mg/kg BMS-933043	0.3 mg/kg BMS-933043	1 mg/kg BMS-933043
Training (day 2)	-3.917	-54.916	-29.043	0.162	1.660
	17.515	24.734	33.714	53.997	-0.048
	29.564	7.488	5.907	18.024	12.620
	-23.042	13.289	23.695	13.466	-13.865
	12.872	-17.384	5.407	-11.337	-7.296
	18.407	14.047	1.825	-17.989	33.864
	5.160	-17.324	25.542	22.940	-5.790
	0.625	18.304	9.515	-27.731	-5.687
	9.129	54.322	22.568	9.896	-5.394
	12.183	5.915	20.966	-23.664	11.280
	37.654	-8.777	-2.211	6.827	-4.256
		1.381	-19.794	-30.548	-17.386
			21.619		-13.115
Mean ± SEM	10.56 ± 4.94	3.42 ± 7.75	9.21 ± 5.10	1.17 ± 7.17	-1.03 ± 3.80
Testing (day 3)	-37.767	-1.495	-24.374	16.697	25.370
	8.182	5.238	58.085	13.188	11.617
	-13.332	30.399	30.161	13.950	14.092
	32.914	-11.785	39.122	32.210	16.034
	-47.755	11.111	34.247	50.151	50.381
	-3.810	60.305	41.627	32.757	36.667
	11.476	6.107	33.604	31.807	20.309
	-1.149	27.678	-1.304	3.353	2.843
	13.580	-36.477	44.255	-9.839	42.802
	29.004	15.251	28.327	0.405	29.506
	-3.311	27.841	32.642	42.771	37.167
		21.506	-6.339	22.038	39.626
			32.254		30.807
Mean ± SEM	-1.09 ± 7.51	12.97 ± 6.98	26.33 ± 6.37	20.79 ± 5.17	27.48 ± 3.86
