# Supplementary material for: The Novel, Nicotinic Alpha7 Receptor Partial Agonist, BMS-933043, Improves Cognition and Sensory Processing in Preclinical Models of Schizophrenia
Source: PLoS One. 2016 Jul 28;11(7):e0159996. doi: 10.1371/journal.pone.0159996 (PMC4965148; doi:10.1371/journal.pone.0159996)
Supplement: S5 Dataset — (PDF) [file pone.0159996.s005.pdf]

S5 Dataset. % Discrimination index (DI) results for individual subjects evaluated in mouse NOR after BMS-933043 treatment (1 - 10 mg/kg).

Session	% Discrimination Index (DI)			
	Vehicle	1 mg/kg BMS-933043	3 mg/kg BMS-933043	10 mg/kg BMS-933043
Training (day 2)	-20.382	37.508	34.831	25.053
	-19.014	15.604	-7.759	-3.752
	-8.403	-13.452	-22.743	-9.240
	11.424	50.637	13.297	16.252
	0.535	20.498	11.713	2.684
	-8.411	2.340	-33.017	15.388
	13.792	-9.246	27.449	3.885
	-7.536	20.111	-22.006	23.627
	-36.006	17.399	-19.297	-4.680
	34.802	7.556	32.852	36.085
		-42.411	-47.820	2.089
		-35.512	-6.703	-15.306
		-27.850		-22.073
Mean ± SEM	-3.92 ± 6.37	3.32 ± 7.75	-3.27 ± 7.83	5.39 ± 4.72
Testing (day 3)	3.672	61.518	79.683	-0.457
	12.588	66.122	-18.950	12.908
	-11.695	48.906	0.957	30.488
	-6.555	10.098	22.321	48.865
	16.645	1.074	28.721	31.973
	-17.283	32.340	13.789	34.568
	9.871	43.933	25.741	44.587
	-1.505	47.930	12.906	23.610
	9.104	7.426	11.878	28.141
	4.454	10.829	78.556	32.466
		36.416	11.870	52.734
		22.876	2.901	41.600
		77.244		51.215
Mean ± SEM	1.93 ± 3.48	35.90 ± 6.77	22.53 ± 8.45	32.28 ± 4.26
