# Supplementary material for: The Novel, Nicotinic Alpha7 Receptor Partial Agonist, BMS-933043, Improves Cognition and Sensory Processing in Preclinical Models of Schizophrenia
Source: PLoS One. 2016 Jul 28;11(7):e0159996. doi: 10.1371/journal.pone.0159996 (PMC4965148; doi:10.1371/journal.pone.0159996)
Supplement: S6 Dataset — (PDF) [file pone.0159996.s006.pdf]

S6 Dataset. % Discrimination index (DI) results for individual subjects evaluated in mouse NOR after treatment with NS-6740 and BMS-933043.

Session	% Discrimination Index (DI)			
	Vehicle/Vehicle	Vehicle/ BMS-933043	NS-6740/ Vehicle	NS-6740/ BMS-933043
Training (day 2)	25.634	-4.818	-6.769	-58.217
	5.242	7.587	15.975	-20.202
	9.271	24.102	-8.023	-31.345
	13.317	-14.844	28.015	12.927
	-2.919	16.037	4.316	-7.767
	-30.568	-57.547	54.554	-28.279
	12.384	10.150	31.082	42.635
	22.909	-38.671	-14.523	7.104
	5.467	-15.602	23.090	-12.240
	58.300	-21.133	12.452	-1.426
	0.000	-4.286	-19.060	31.444
	7.115	0.310	-13.043	-10.934
	-71.62	14.545	-20.904	5.412
	7.490	-8.986	0.722	-31.737
	5.703	5.131	8.607	30.337
	-11.449			
Mean ± SEM	3.53 ± 6.82	-5.87 ± 5.59	6.43 ± 5.54	-4.82 ± 7.09
Testing (day 3)	8.146	26.274	18.155	0.898
	4.055	56.605	-8.110	-16.024
	13.622	36.560	0.319	21.509
	-19.231	28.407	-10.083	19.058
	26.183	33.003	10.541	-0.179
	-4.303	22.241	-2.851	-24.362
	41.338	25.363	-13.536	38.373
	2.360	1.070	24.696	11.786
	39.992	38.444	-46.553	9.968
	-34.261	9.918	18.258	3.278
	-4.753	49.569	-7.893	12.335
	27.167	21.604	1.673	-4.193
	-12.008	31.363	1.887	-20.495
	38.071	36.528	5.935	5.243
	-0.874	51.892	-7.708	0.282
	-8.257			
Mean ± SEM	7.33 ± 5.55	31.26 ± 3.86	-1.02 ± 4.40	3.83 ± 4.25
