# Supplementary material for: The Novel, Nicotinic Alpha7 Receptor Partial Agonist, BMS-933043, Improves Cognition and Sensory Processing in Preclinical Models of Schizophrenia
Source: PLoS One. 2016 Jul 28;11(7):e0159996. doi: 10.1371/journal.pone.0159996 (PMC4965148; doi:10.1371/journal.pone.0159996)
Supplement: S7 Dataset — (PDF) [file pone.0159996.s007.pdf]

S7 Dataset. Number of trials to criteria for individual subjects evaluated in the MK-801 set shift model after treatment with BMS-933043.

Study	Vehicle/ Vehicle	Vehicle/ MK-801	0.3 mg/kg BMS-933043 /MK-801	1 mg/kg BMS-933043 /MK-801	3 mg/kg BMS-933043 /MK-801
BMS-933043 0.3 - 3 mg/kg	38	66	80	57	61
	20	80	57	25	42
	50	71	78	65	40
	66	75	67	53	64
	49	66	45	60	44
	61	61	70	69	55
	50	67	66	42	48
	49	60	70	63	26
	32	78	62	73	50
		80	69	37	73
Mean ± SEM	46.1 ± 4.7	70.4 ± 2.4	66.4 ± 3.2	54.4 ± 4.8	50.3 ± 4.3
Study	Vehicle/ Vehicle	Vehicle/ MK-801	3 mg/kg BMS-933043 /MK-801	10 mg/kg BMS-933043 /MK-801	30 mg/kg BMS-933043 /MK-801
BMS-933043 3 - 30 mg/kg	63	64	59	50	55
	61	52	51	63	44
	76	80	80	72	80
	58	72	28	49	62
	63	75	67	50	80
	42	75	54	60	80
	46	66	20	51	80
	50	80	36	49	55
	32	71	37	61	76
	36	71	51	35	
Mean ± SEM	52.7 ± 4.4	70.6 ± 2.6	48.3 ± 5.8	54.0 ± 3.2	68.0 ± 4.7
