# Supplementary material for: The Novel, Nicotinic Alpha7 Receptor Partial Agonist, BMS-933043, Improves Cognition and Sensory Processing in Preclinical Models of Schizophrenia
Source: PLoS One. 2016 Jul 28;11(7):e0159996. doi: 10.1371/journal.pone.0159996 (PMC4965148; doi:10.1371/journal.pone.0159996)
Supplement: S8 Dataset — (PDF) [file pone.0159996.s008.pdf]

S8 Dataset. % Time spent in the novel arm for individual mice evaluated in the MK-801 Y maze procedure after treatment with BMS-933043.

	Vehicle/ Vehicle	Vehicle/ MK-801	0.03 mg/kg BMS-933043/ MK-801	0.1 mg/kg BMS-933043/ MK-801	0.3 mg/kg BMS-933043/ MK-801	1 mg/kg BMS-933043/ MK-801	3 mg/kg BMS-933043/ MK-801	10 mg/kg BMS-933043/ MK-801
	87	85	22	59	8	58	58	58
	79	58	79	79	69	82	77	65
	73	67	68	78	56	86	59	84
	71	47	90	29	49	84	86	63
	84	50	89	46	90	91	90	69
	55	20	28	76	65	88	91	69
	85	54	69	87	77	76	60	80
	71	51	74	85	59	75	78	89
	59	23	69	37	62	75	71	65
	77	11	65	73	66	54	73	63
	60	84	27	53	61	57	70	77
	76	23	80	55	64	67	84	91
	86	51	9	57	77	78	86	77
	72	74	14	53	56	87	93	71
	77	40	62	36	41	67	61	87
	86	81	81	49	83	86	71	58
	80		87	27	54			79
	77			67				67
Mean ± SEM	75.3 ± 2.2	51.2 ± 5.9	59.6 ± 6.8	58.1 ± 4.4	61.0 ± 4.5	75.7 ± 3.0	75.5 ± 3.0	72.8 ± 2.5
