# Supplementary material for: The Novel, Nicotinic Alpha7 Receptor Partial Agonist, BMS-933043, Improves Cognition and Sensory Processing in Preclinical Models of Schizophrenia
Source: PLoS One. 2016 Jul 28;11(7):e0159996. doi: 10.1371/journal.pone.0159996 (PMC4965148; doi:10.1371/journal.pone.0159996)
Supplement: S9 Dataset — (PDF) [file pone.0159996.s009.pdf]

S9 Dataset. Gating ratio for individual subjects evaluated in the S(+)ketamine N40 gating model after treatment with BMS-933043.

Treatment	Vehicle		BMS-933043 0.3 mg/kg	
Gating Ratio	Baseline	Post-Ketamine	Baseline	Post-Ketamine
	0.57	0.80	0.33	0.65
	0.42	0.78	0.47	0.59
	0.44	1.25	0.27	0.45
	0.76	1.50	0.70	0.83
	0.37	0.90	0.37	0.65
	0.58	1.00	0.44	1.74
	0.87	1.10	0.89	0.83
	0.25	0.57	0.55	0.91
	0.52	0.50	0.33	0.75
	0.37	0.65	0.37	1.02
	0.60	1.23	0.51	0.40
	0.54	1.44	0.49	0.58
	0.29	1.00	0.61	0.43
Mean ± SEM	0.51 ± 0.049	0.98 ± 0.088	0.49 ± 0.048	0.76 ± 0.097
Treatment	Vehicle		BMS-933043 0.56 mg/kg	
Gating Ratio	Baseline	Post-Ketamine	Baseline	Post-Ketamine
	0.31	1.06	0.48	0.63
	0.72	1.56	0.55	1.18
	0.56	1.62	0.35	0.48
	0.41	1.04	0.47	0.82
	0.49	1.27	0.50	0.85
	0.86	1.00	0.33	0.95
	0.84	1.82	0.52	0.69
	0.37	1.21	0.39	0.68
	0.38	2.68	0.43	0.41
	0.34	1.41	0.38	0.92
	0.44	2.07	0.53	0.86
	0.71	1.17	0.44	1.08
	0.49	0.96	0.62	0.48
Mean ± SEM	0.53 ± 0.053	1.5 ± 0.14	0.46 ± 0.023	0.77 ± 0.065
Treatment	Vehicle		BMS-933043 1 mg/kg	
Gating Ratio	Baseline	Post-Ketamine	Baseline	Post-Ketamine
	0.62	1.13	0.38	1.00
	0.39	1.24	0.51	0.35
	0.49	1.50	0.19	1.36
	0.33	0.62	0.47	1.00
	0.51	1.24	0.27	0.56
	0.73	1.35	0.45	0.50
	0.44	1.90	0.55	1.01
	0.12	1.39	0.30	0.64
	0.25	2.72	0.48	1.15
	0.41	1.31	0.17	0.45
	0.87	1.31	0.38	0.88
	0.65	1.24	0.50	1.65

	0.56	2.12	0.64	0.53
Mean ± SEM	0.49 ± 0.056	1.5 ± 0.14	0.41 ± 0.039	0.85 ± 0.11
Treatment	Vehicle		BMS-933043 3 mg/kg	
Gating Ratio	Baseline	Post-Ketamine	Baseline	Post-Ketamine
	0.49	2.45	0.46	1.11
	0.31	0.56	0.31	0.48
	0.62	0.75	0.65	0.58
	0.39	1.64	0.81	0.76
	0.24	1.07	1.02	0.35
	0.31	0.91	0.39	0.32
	0.32	0.73	0.48	0.91
	0.34	0.80	0.51	1.31
	0.44	1.06	0.34	0.76
	0.36	1.24	0.53	0.90
	0.42	0.73	0.58	0.53
	0.65	1.18	0.54	0.86
	0.59	0.98	0.57	0.70
Mean ± SEM	0.42 ± 0.036	1.1 ± 0.14	0.55 ± 0.053	0.74 ± 0.08
Treatment	Vehicle		BMS-933043 10 mg/kg	
Gating Ratio	Baseline	Post-Ketamine	Baseline	Post-Ketamine
	0.53	0.87	0.55	0.43
	0.51	0.77	0.37	1.54
	0.15	0.75	0.17	0.44
	0.31	0.91	0.49	0.93
	0.43	1.52	0.32	0.69
	0.25	0.58	0.31	0.85
	0.42	1.35	0.57	1.55
	0.36	1.31	0.46	0.96
	0.52	1.05	0.56	0.35
	0.49	1.74	0.49	1.44
	0.45	0.79	0.57	0.57
	0.32	1.64	0.37	0.50
Mean ± SEM	0.4 ± 0.034	1.1 ± 0.11	0.44 ± 0.037	0.85 ± 0.13
