# Supplementary material for: The Novel, Nicotinic Alpha7 Receptor Partial Agonist, BMS-933043, Improves Cognition and Sensory Processing in Preclinical Models of Schizophrenia
Source: PLoS One. 2016 Jul 28;11(7):e0159996. doi: 10.1371/journal.pone.0159996 (PMC4965148; doi:10.1371/journal.pone.0159996)
Supplement: S10 Dataset — (PDF) [file pone.0159996.s010.pdf]

S10 Dataset. Number of trials to criteria at each discrimination stage for individual rats evaluated in the neonatal PCP ID/ED model.

Discrimination	Sham/ Vehicle	PCP/ Vehicle	PCP/ 0.1 mg/kg BMS-933043	PCP/ 0.3 mg/kg BMS-933043	PCP/ 1 mg/kg BMS-933043	PCP/ 3 mg/kg BMS-933043
Simple	7	16	7	10	6	7
	15	8	11	15	12	6
	8	11	7	13	13	7
	14	7	9	8	6	7
	7	7	8	9	12	16
	9	7	10	12	11	7
	9	14	7	6	8	9
	7	11	8	19	9	16
	8	8	6	7	6	6
	7	14	16	8		
Complex	11	8	6	18	6	6
	11	6	16	10	11	6
	7	13	7	7	11	8
	14	10	8	6	7	16
	19	16	21	10	7	10
	13	8	19	8	13	15
	13	8	8	6	6	17
	10	6	7	16	14	11
	6	11	6	6	14	10
	11	16	6	8		
Intra-Dimensional Shift 1	9	6	8	10	7	13
	15	11	6	6	8	11
	6	10	11	7	11	18
	19	6	7	11	13	7
	15	10	10	8	6	13
	12	13	6	7	16	10
	12	14	7	7	6	9
	7	9	6	10	13	11
	7	7	7	15	8	7
	7	13	8	7		
Intra-Dimensional Shift 2	9	9	9	7	7	7
	9	11	7	8	8	8
	6	10	6	10	8	9
	7	10	11	7	9	8
	7	9	11	9	6	7
	12	7	8	6	14	13
	9	9	9	7	10	8
	6	12	7	10	9	7
	6	6	8	12	8	11
	6	9	8	8		

Intra-Dimensional Shift 2 Reversal	9	9	7	6	10	7
	9	8	8	6	11	8
	10	12	10	8	8	8
	6	7	6	7	9	9
	11	7	11	13	7	11
	10	8	9	8	9	6
	7	6	8	10	6	10
	6	7	9	11	10	10
	11	8	6	9	7	10
	11	13	10	10		
Extra-Dimensional Shift	6	22	15	10	13	14
	13	29	8	16	17	9
	12	14	12	9	17	10
	10	19	9	19	10	15
	7	23	11	10	8	12
	10	29	18	6	7	8
	9	24	14	11	10	9
	13	28	19	7	7	8
	7	12	8	8	11	6
	6	28	17	6		
