# Supplementary material for: The Novel, Nicotinic Alpha7 Receptor Partial Agonist, BMS-933043, Improves Cognition and Sensory Processing in Preclinical Models of Schizophrenia
Source: PLoS One. 2016 Jul 28;11(7):e0159996. doi: 10.1371/journal.pone.0159996 (PMC4965148; doi:10.1371/journal.pone.0159996)
Supplement: S11 Dataset — (PDF) [file pone.0159996.s011.pdf]

S11 Dataset. Individual AUC (150-200 ms) results for neonatal PCP treated rats evaluated in the MMN model after treatment with BMS-933043.

Study 1	Vehicle	0.01 mg/kg BMS-933043	0.1 mg/kg BMS-933043
	-0.000040	0.000184	-0.000065
	-0.000084	-0.000213	0.000032
	-0.000259	0.000152	0.000006
	0.000415	-0.000150	-0.000071
	-0.000090	-0.000085	-0.000085
	-0.000211	0.000079	0.000006
	-0.000092	-0.000170	-0.000297
	0.000101	-0.000173	-0.000118
	-0.000215	-0.000105	-0.000262
	0.000019	-0.000192	-0.000160
	-0.000156	-0.000124	-0.000175
	0.000023	-0.000002	-0.000225
	-0.000245	-0.000060	-0.000130
Mean	-6.415e-005	-6.608e-005	-0.0001188
SEM	5.046e-005	3.645e-005	2.873e-005
Study 2	Vehicle	0.03 mg/kg BMS-933043	0.1 mg/kg BMS-933043
	-0.000148	-0.000686	-0.000204
	0.000000	-0.000173	-0.000978
	0.000189	-0.000246	-0.000092
	0.000032	-0.000095	-0.000137
	-0.000133	-0.000251	-0.000114
	-0.000195	-0.000307	0.000051
	-0.000264	-0.000162	-0.000212
	-0.000195	0.000020	-0.000220
	-0.000026	-0.000202	0.000149
	-0.000219	-0.000108	-0.000062
	0.000114	-0.000463	-0.000099
	-0.000097	-0.000161	-0.000140
Mean	-7.850e-005	-0.0002362	-0.0001715
SEM	4.075e-005	5.347e-005	7.962e-005
Study 3	Vehicle	1 mg/kg BMS-933043	3 mg/kg BMS-933043
	-0.000093	-0.000135	-0.000308
	-0.000102	-0.000245	0.000042
	-0.000002	-0.000227	-0.000215
	0.000195	-0.000008	-0.000196
	0.000095	-0.000041	-0.000025
	-0.000217	-0.000308	-0.000092
	-0.000270	-0.000125	0.000040
	0.000141	0.000174	-0.000148
	0.000057	-0.000206	-0.000204
	-0.000233	-0.000100	-0.000211
	-0.000063	-0.000195	0.000128
	0.000138	-0.000135	-0.000285
	-0.000168	-0.000516	-0.000269
Mean	-4.015e-005	-0.000159	-0.0001341
SEM	4.340e-005	4.519e-005	3.892e-005
