# Supplementary material for: The Novel, Nicotinic Alpha7 Receptor Partial Agonist, BMS-933043, Improves Cognition and Sensory Processing in Preclinical Models of Schizophrenia
Source: PLoS One. 2016 Jul 28;11(7):e0159996. doi: 10.1371/journal.pone.0159996 (PMC4965148; doi:10.1371/journal.pone.0159996)
Supplement: S12 Dataset — (PDF) [file pone.0159996.s012.pdf]

S12 Dataset. Ex vivo α7 nAChR receptor occupancy results for individual subjects.

**Mouse Dose Response**	**Individual % occupancy result**
0.3 mg/kg sc BMS-933043	11.2, -5.2, 7.5, -6.3
1 mg/kg sc BMS-933043	23.9, 18.3, 25.5, 25.4
3 mg/kg sc BMS-933043	45.1, 41.3, 56.2, 44.9
10 mg/kg sc BMS-933043	78.5, 76.4, 74.8, 75.4
100 mg/kg sc BMS-933043	91.3, 93.3, 94.1, 92.1
**Rat Dose Response**	**Individual % occupancy results**
1 mg/kg po BMS-933043	27, 22, 6, 42
3 mg/kg po BMS-933043	65, 63, 62, 28
10 mg/kg po BMS-933043	80, 83, 78, 77
30 mg/kg po BMS-933043	82, 90, 67, 69
