# Supplementary material for: The Novel, Nicotinic Alpha7 Receptor Partial Agonist, BMS-933043, Improves Cognition and Sensory Processing in Preclinical Models of Schizophrenia
Source: PLoS One. 2016 Jul 28;11(7):e0159996. doi: 10.1371/journal.pone.0159996 (PMC4965148; doi:10.1371/journal.pone.0159996)

S1 Fig. Representative mean evoked response potential waveforms and calculation of the AUC to determine treatment effects on MMN in neonatal PCP treated rats. Results show the group mean evoked response potential (ERP; mV) recorded over time (s) prior to and after presentation of standard (blue line) and deviant (red line) tones and the difference waveform (ie MMN, green line). Tone onset is shown by the black bar. The hatched rectangle indicates the period of the difference waveform used to calculate the AUC for each subject.

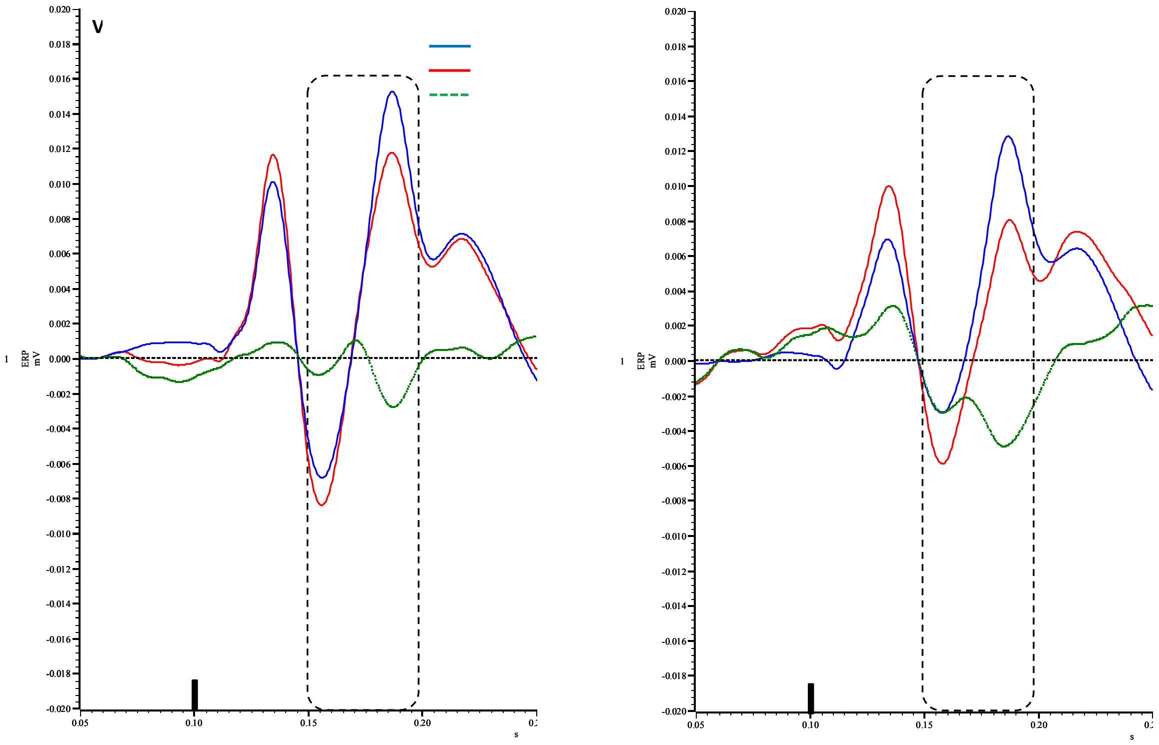

Supplement: S1 Fig — (PDF) [file pone.0159996.s013.pdf]
