# Supplementary material for: The Novel, Nicotinic Alpha7 Receptor Partial Agonist, BMS-933043, Improves Cognition and Sensory Processing in Preclinical Models of Schizophrenia
Source: PLoS One. 2016 Jul 28;11(7):e0159996. doi: 10.1371/journal.pone.0159996 (PMC4965148; doi:10.1371/journal.pone.0159996)

S2 Fig. Inhibition of [^{3}H]A-585539 binding to α7 nACh receptors. Representative competition binding curves for BMS-933043, EVP-6124, A-582941 and (-)-nicotine at A) native rat brain α7 nAChR and B) recombinant human α7 nAChR. All data points are the mean ± S.E.M. determined from duplicate samples at each concentration.

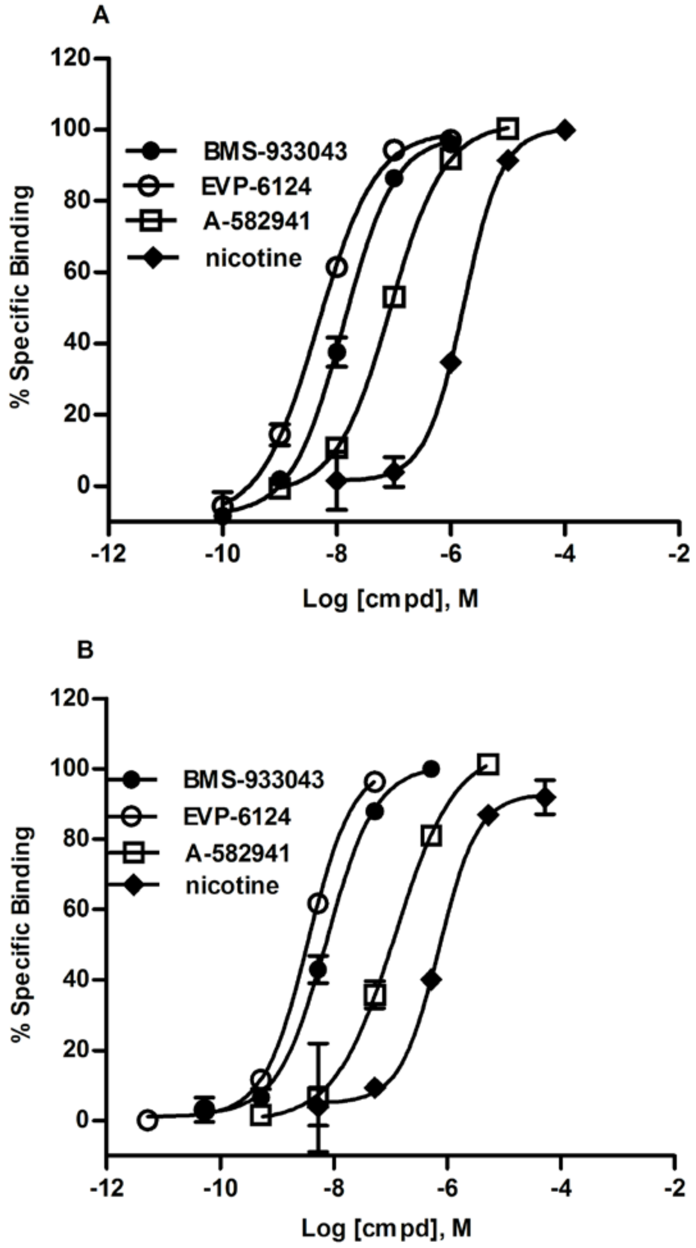

Supplement: S2 Fig — (PDF) [file pone.0159996.s014.pdf]
