# Supplementary material for: The Novel, Nicotinic Alpha7 Receptor Partial Agonist, BMS-933043, Improves Cognition and Sensory Processing in Preclinical Models of Schizophrenia
Source: PLoS One. 2016 Jul 28;11(7):e0159996. doi: 10.1371/journal.pone.0159996 (PMC4965148; doi:10.1371/journal.pone.0159996)

S3 Fig. Inhibition of [^{3}H]-granisetron binding to human 5-HT_{3A} receptors. Representative competition binding curves for BMS-933043, granisetron, MDL-72222 and EVP-6124. Each point shows the mean ± S.E.M. determined from duplicate samples at each concentration.

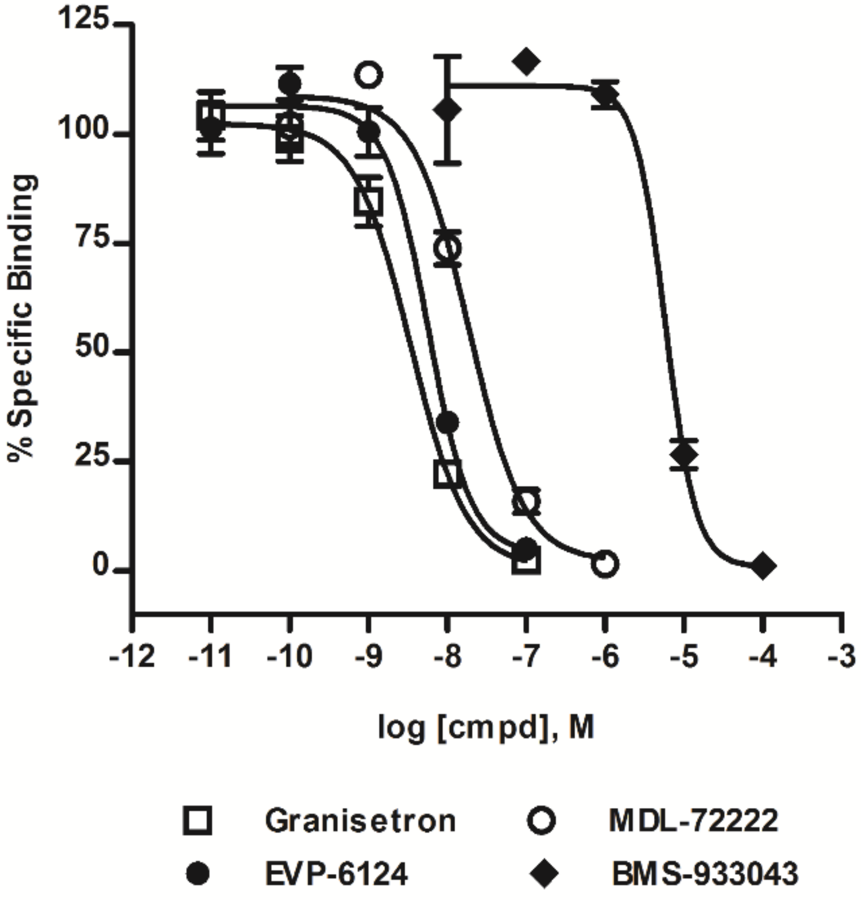

Supplement: S3 Fig — (PDF) [file pone.0159996.s015.pdf]
