# Supplementary material for: The Novel, Nicotinic Alpha7 Receptor Partial Agonist, BMS-933043, Improves Cognition and Sensory Processing in Preclinical Models of Schizophrenia
Source: PLoS One. 2016 Jul 28;11(7):e0159996. doi: 10.1371/journal.pone.0159996 (PMC4965148; doi:10.1371/journal.pone.0159996)

S4 Fig. Agonist profile of EVP-6124 and TC-5619 in voltage clamp electrophysiology. Results show the mean ± S.E.M. peak current (open circles) or net charge crossing the membrane (closed circles) normalized to the maximal response induced by ACh determined in HEK293/rat α7 nAChR cells (TC-5619; n=3) or HEK293/human α7 nAChR cells (EVP-6124; n=4).

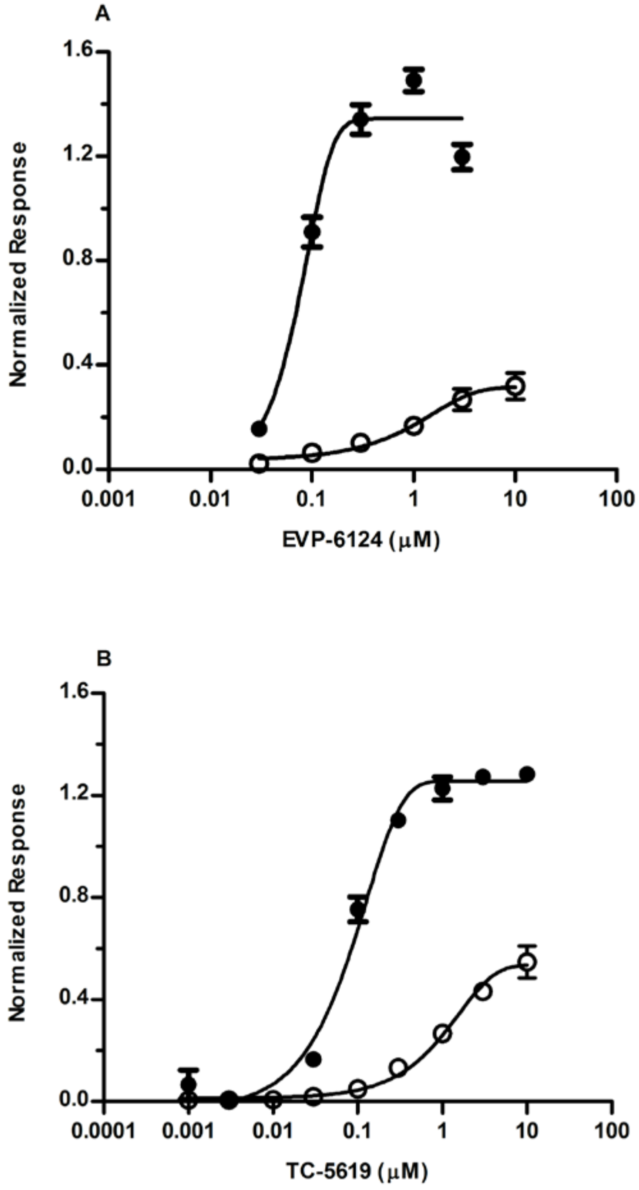

Supplement: S4 Fig — (PDF) [file pone.0159996.s016.pdf]
