# Supplementary material for: The Novel, Nicotinic Alpha7 Receptor Partial Agonist, BMS-933043, Improves Cognition and Sensory Processing in Preclinical Models of Schizophrenia
Source: PLoS One. 2016 Jul 28;11(7):e0159996. doi: 10.1371/journal.pone.0159996 (PMC4965148; doi:10.1371/journal.pone.0159996)
Supplement: S5 Fig — (PDF) [file pone.0159996.s017.pdf]

S5 Fig. Treatment with BMS-933043 does not alter total object exploration time in the training session or testing session in mice. Results show the mean ± SEM total object exploration time (s) in mice treated with vehicle, BMS-933043 or NS-6740 prior to BMS-933043 treatment (n=10-16/group). Results from 2 way repeated measures analysis of variance show that exploration time is not significantly different across any of the treatment conditions but is lower in the test session compared to training.

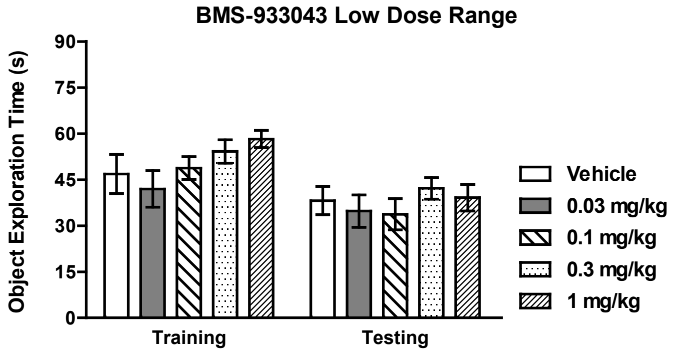

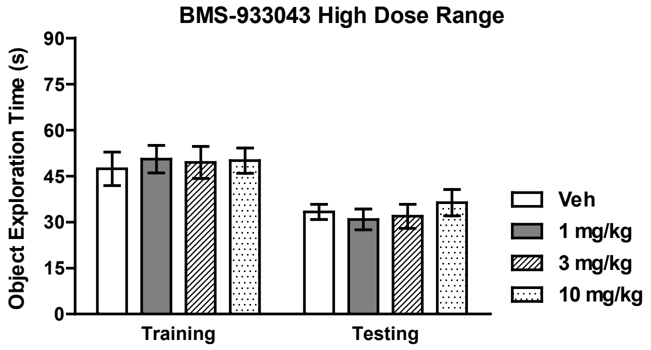

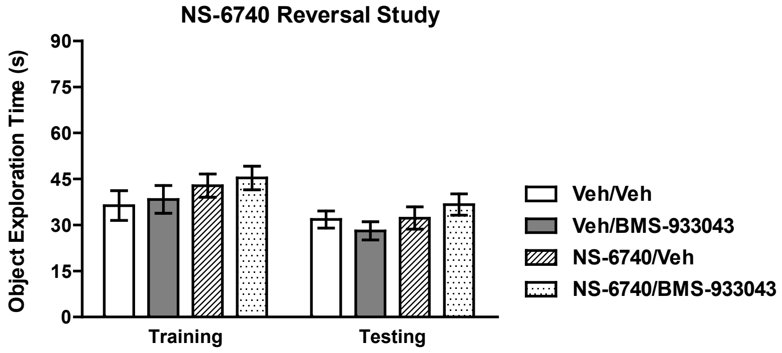

ANOVA results: BMS-933043 low dose range: treatment effect $F(4,56)=1.3$, $p=0.28$; session effect $F(1,56)=34.88$, $P<0.0001$; treatment x session interaction $F(4,56)=1.08$, $p=0.38$. BMS-933043 high dose range: treatment effect $F(3,44)=0.12$, $p=0.94$; session effect $F(1,44)=71.75$, $p<0.0001$; treatment x session interaction $F(3,44)=0.59$, $p=0.63$. NS-6740 reversal study: treatment effect $F(3,57)=1.6$, $p=0.2$; session effect $F(1,57)=11.14$, $p=0.0015$; treatment x session interaction $F(3,57)=0.31$, $p=0.82$.
