# Supplementary material for: The Novel, Nicotinic Alpha7 Receptor Partial Agonist, BMS-933043, Improves Cognition and Sensory Processing in Preclinical Models of Schizophrenia
Source: PLoS One. 2016 Jul 28;11(7):e0159996. doi: 10.1371/journal.pone.0159996 (PMC4965148; doi:10.1371/journal.pone.0159996)

S6 Fig. NS-6740 does not impact NOR performance at doses achieving high levels of α7nAChR occupancy in mice. A) Mice were treated with either vehicle or NS-6740 40 min prior to training and NOR examined 24 h later. Results show the mean ± S.E.M. time spent exploring the novel (white bar) or familiar (grey bar) objects (n=10-14/group). B) Mice were treated with NS-6740 and brains collected for occupancy determinations 30 min later. Results show the mean ± SEM occupancy (n=4/group).

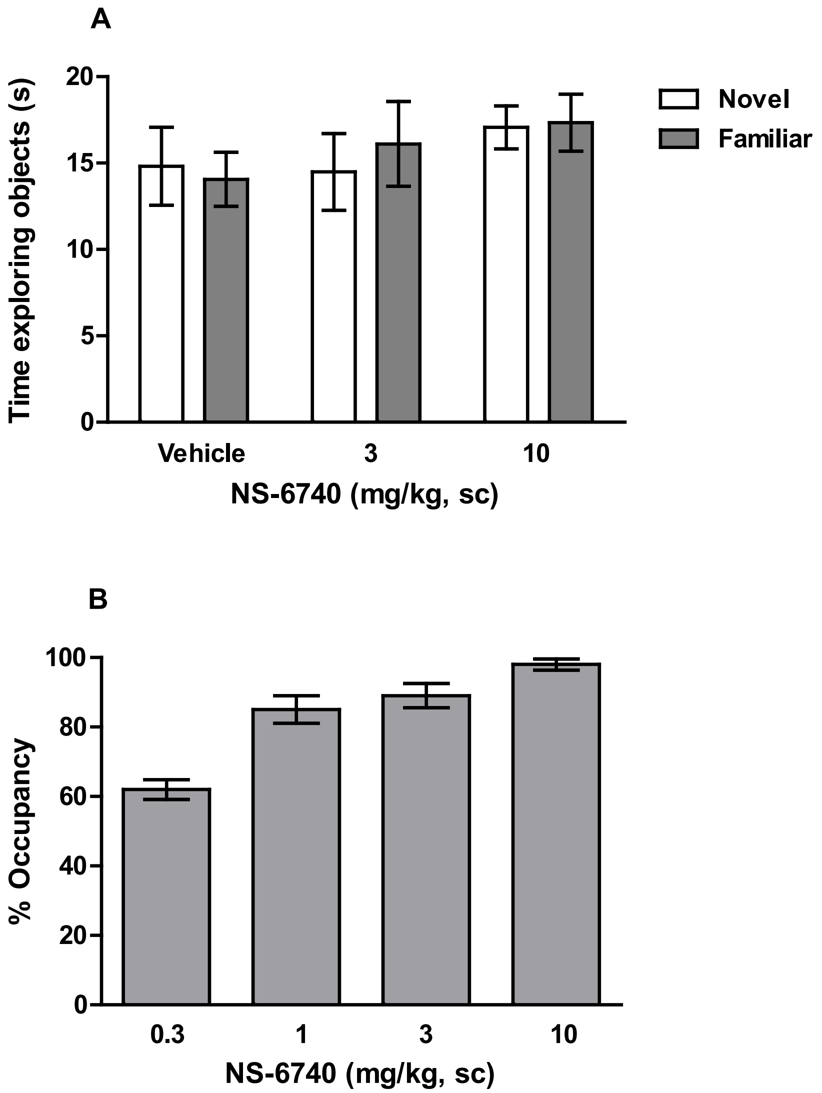

Supplement: S6 Fig — (PDF) [file pone.0159996.s018.pdf]
