# Supplementary material for: The Novel, Nicotinic Alpha7 Receptor Partial Agonist, BMS-933043, Improves Cognition and Sensory Processing in Preclinical Models of Schizophrenia
Source: PLoS One. 2016 Jul 28;11(7):e0159996. doi: 10.1371/journal.pone.0159996 (PMC4965148; doi:10.1371/journal.pone.0159996)
Supplement: S7 Fig — (PDF) [file pone.0159996.s019.pdf]

S7 Fig. BMS-933043 reduces perseverative errors in MK-801-treated rats performing the maze based set shifting task. Results show the mean ± S.E.M. number of perseverative errors and were analyzed by ANOVA followed by Dunnett's post hoc analysis comparing all groups to vehicle/MK-801-treated rats; * p<0.05, ** p<0.01, *** p<0.001 (n=9-10/group).

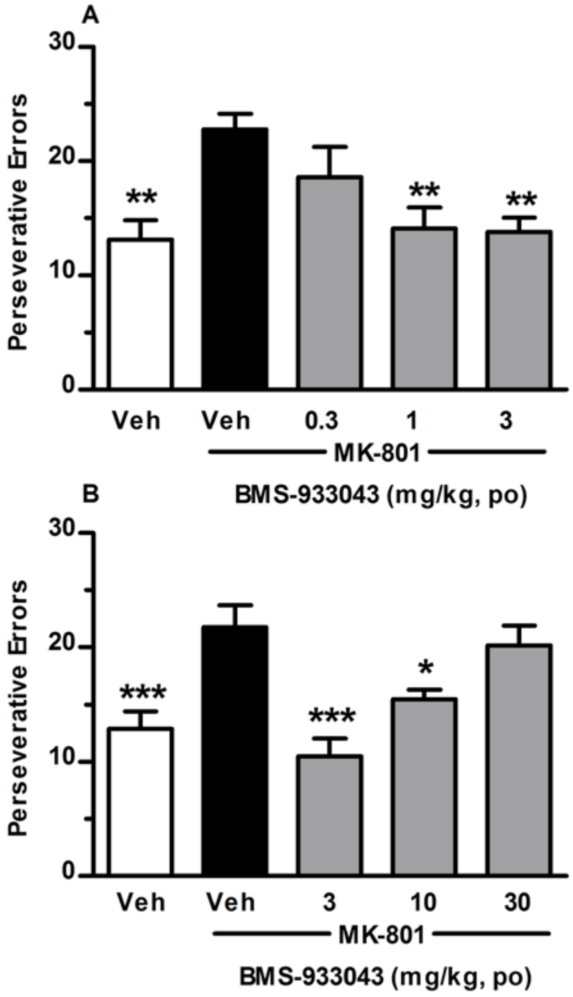

ANOVA results: A) F(4,44)=5.09, p=0.002; B) F(4,44)=9.475, p<0.0001
