# Supplementary material for: The Novel, Nicotinic Alpha7 Receptor Partial Agonist, BMS-933043, Improves Cognition and Sensory Processing in Preclinical Models of Schizophrenia
Source: PLoS One. 2016 Jul 28;11(7):e0159996. doi: 10.1371/journal.pone.0159996 (PMC4965148; doi:10.1371/journal.pone.0159996)
Supplement: S8 Fig — (PDF) [file pone.0159996.s020.pdf]

S8 Fig. EDS impairment and improvement following BMS-933043 treatment is similar in neonatal PCP treated rats shifted from media to odor or odor to media at the EDS stage. Results show the mean ± SEM trials to achieve performance criteria at each discrimination stage (n= 9-10/group) and were analyzed by 2 way repeated measures ANOVA followed by Dunnett's post hoc test; *** p=0.0004, **** p=0.0001 versus PCP/veh.

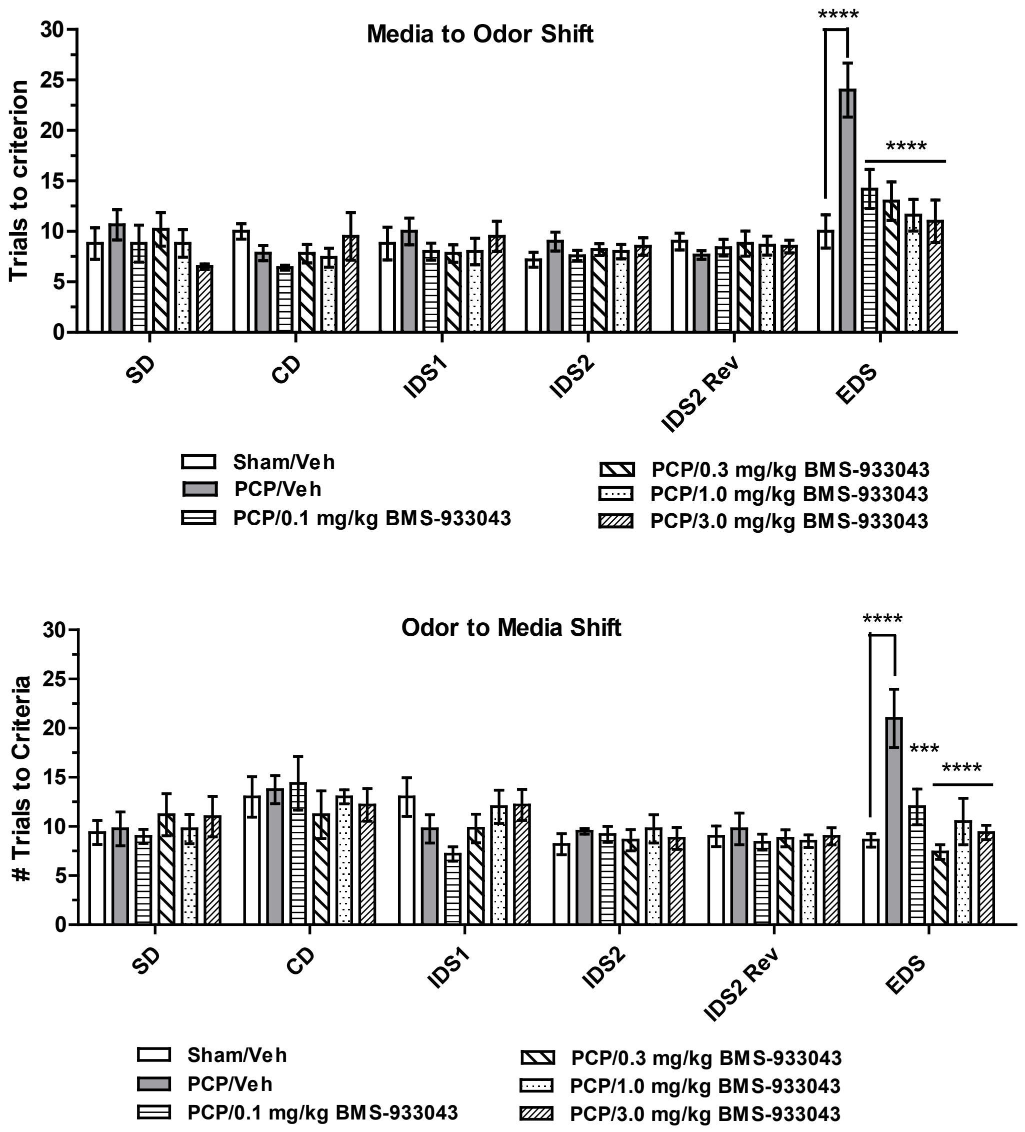

ANOVA results: Media to odor: Treatment effect $F(5,24)=3.43$, $p=0.018$; Discrimination effect $F(5,120)=18.17$, $p<0.0001$; treatment x discrimination interaction $F(25,120)=3.27$, $p<0.0001$. Odor to media: treatment effect $F(5,22)=1.42$, $p=0.257$; Discrimination effect $F(5,110)=6.637$, $p<0.0001$; treatment x discrimination interaction $F(25,110)=2.325$, $p=0.0015$.
