# Supplementary material for: The Novel, Nicotinic Alpha7 Receptor Partial Agonist, BMS-933043, Improves Cognition and Sensory Processing in Preclinical Models of Schizophrenia
Source: PLoS One. 2016 Jul 28;11(7):e0159996. doi: 10.1371/journal.pone.0159996 (PMC4965148; doi:10.1371/journal.pone.0159996)
Supplement: S9 Fig — (PDF) [file pone.0159996.s021.pdf]

S9 Fig. BMS-933043 decreases the time to complete the EDS discrimination in neonatal PCP-treated rats. Results show the mean ± S.E.M. time to complete each discrimination and were analyzed by 2 way repeated measures ANOVA followed by Dunnett's post-hoc analysis comparing all groups to neonatal PCP/vehicle treated rats; *p=0.0147, *** p=0.0005, ****p = 0.0001 (n= 9-10/group).

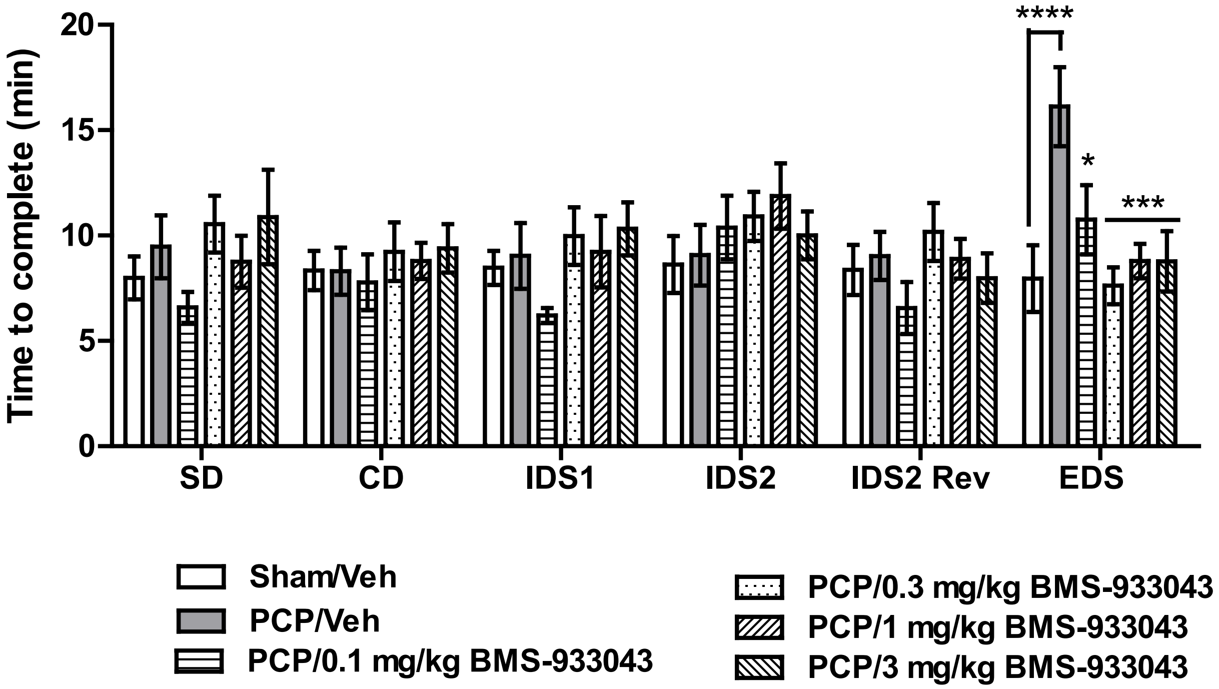

ANOVA results: Treatment effect F(5,52)=1.041, p=0.4036; Discrimination effect F(5,260)=2.469, p=0.033; treatment x discrimination interaction F(25,260)=2.356, p=0.0004.
