# Supplementary material for: The Novel, Nicotinic Alpha7 Receptor Partial Agonist, BMS-933043, Improves Cognition and Sensory Processing in Preclinical Models of Schizophrenia
Source: PLoS One. 2016 Jul 28;11(7):e0159996. doi: 10.1371/journal.pone.0159996 (PMC4965148; doi:10.1371/journal.pone.0159996)
Supplement: S1 Table — (PDF) [file pone.0159996.s022.pdf]

S1 Table. Example of exemplars used at each discrimination phase of the rat ID/ED task.

Discrimination Stage	Correct Dimension	Correct Exemplar	Exemplars Presented	
			Medium	Odor
SD	Medium	Foam rubber	Foam rubber<br>Plastic beads	None
CD	Medium	Foam rubber	Foam rubber<br>Plastic beads	Vanilla<br>Jasmine
IDS1	Medium	Yarn	Yarn<br>Pom Poms	Orange<br>Sage
IDS2	Medium	Glass beads	Glass beads<br>Aquarium gravel	Patchouli<br>Strawberry
IDSrev	Medium	Aquarium gravel	Glass beads<br>Aquarium gravel	Patchouli<br>Strawberry
EDS	Odor	Cinnamon	Aspen shavings<br>Shredded Folder	Cinnamon<br>Gardenia

SD: simple discrimination; CD: compound discrimination; IDS1: intra-dimensional shift 1; IDS2: intra-dimensional shift 2; IDS2rev: intra-dimensional shift 2 reversal; EDS: extra-dimensional shift.
