# Supplementary material for: The Novel, Nicotinic Alpha7 Receptor Partial Agonist, BMS-933043, Improves Cognition and Sensory Processing in Preclinical Models of Schizophrenia
Source: PLoS One. 2016 Jul 28;11(7):e0159996. doi: 10.1371/journal.pone.0159996 (PMC4965148; doi:10.1371/journal.pone.0159996)
Supplement: S2 Table — (PDF) [file pone.0159996.s023.pdf]

S2 Table. Pharmacological evaluation of BMS-933043 at other receptor and enzyme targets.

Target	Species	Assay type	Result
Acetylcholinesterase	Eel	Enzyme	IC_{50} >60 μM
Adenosine A2a	Human	Binding	IC_{50} >30 μM
Adrenergic α1B	Human	Binding	IC_{50} >30 μM
Adrenergic α1D	Human	Binding	IC_{50} >30 μM
Adrenergic α2A	Human	Binding	IC_{50} >30 μM
Adrenergic α2C	Human	Binding	IC_{50} >30 μM
Androgen receptor	Rat	Binding	IC_{50} >150 μM
Nav1.5	Human	Functional	IC_{50} >30 μM
CB1	Human	Binding	-5% inhibition @ 10 μM
Dopamine D1	Human	Binding	IC_{50} >30 μM
Dopamine D2	Human	Binding	IC_{50} >30 μM
Dopamine transporter	Human	Binding	IC_{50} >30 μM
Estrogen receptor alpha	Human	Binding	IC_{50} >150 μM
GABA-A $\alpha1\beta2\gamma$ 2	Human	Functional	IC_{50} >30 μM
Glucocorticoid receptor	Human	Binding	IC_{50} >150 μM
Histamine H1	Human	Binding	IC_{50} >30 μM
Histamine H2	Human	Binding	IC_{50} >30 μM
L-type calcium channel	Human	Functional	IC_{50} >25 μM
MAO-A	Human	Enzyme	IC_{50} >30 μM
MAO-B	Human	Enzyme	IC_{50} >30 μM
Muscarinic M_1	Human	Binding	11% inhibition @ 10 μM
Muscarinic M_2	Human	Binding	IC_{50} = 19.14 μM
Muscarinic M_3	Human	Binding	18% inhibition @ 10 μM
Muscarinic M_4	Human	Binding	10% inhibition @ 10 μM
Muscarinic M_5	Human	Binding	8% inhibition @ 10 μM
NMDA NR1/2A	Human	Functional	IC_{50} >30 μM
Norepinephrine transporter	Human	Binding	IC_{50} >30 μM
Opiate Mu	Human	Binding	IC_{50} >30 μM
Opiate Kappa	Human	Binding	IC_{50} >30 μM
PDE3	Human	Enzyme	IC_{50} >30 μM
PDE4	Human	Enzyme	IC_{50} >30 μM
Progesterone receptor	Human	Binding	IC_{50} >150 μM
5-HT_{1A}	Human	Binding	9% inhibition @ 10 μM
5-HT_{1B}	Rat	Binding	0% inhibition @ 10 μM
5-HT_{2A}	Human	Functional	EC_{50} >10 μM
5-HT_{2B}	Human	Functional	EC_{50} >10 μM
5-HT transporter	Human	Binding	IC_{50} >30 μM
