# Supplementary material for: The Novel, Nicotinic Alpha7 Receptor Partial Agonist, BMS-933043, Improves Cognition and Sensory Processing in Preclinical Models of Schizophrenia
Source: PLoS One. 2016 Jul 28;11(7):e0159996. doi: 10.1371/journal.pone.0159996 (PMC4965148; doi:10.1371/journal.pone.0159996)
Supplement: S3 Table — (PDF) [file pone.0159996.s024.pdf]

S3 Table. Plasma and brain concentrations of BMS-933043 determined after subcutaneous or oral dosing in rodents.

Model	BMS-933043 Dose & Route	Time	Plasma (nM) Mean ± SD	Brain (nM) Mean ± SD	Brain/Plasma (%)
Mouse NOR	0.03 mg/kg sc	30 min	14 ± 2.7	<LLQ	ND
	0.10 mg/kg sc	30 min	52 ± 3.6	<LLQ	ND
	0.30 mg/kg sc	30 min	213 ± 49	24 ± 3.9	12%
	1 mg/kg sc	30 min	719 ± 69	78 ± 5.2	11%
Mouse NOR	1 mg/kg sc	30 min	459 ± 59	97 ± 16	21%
	3 mg/kg sc	30 min	1,568 ± 103	297 ± 48	19%
	10 mg/kg sc	30 min	5,555 ± 789	1,113 ± 162	20%
Rat MK-801 Set Shift	0.30 mg/kg po	150 min	5.7 ± 0.9	<LLQ	ND
	1 mg/kg po	150 min	50 ± 7.4	13 ± 3	25%
	3 mg/kg po	150 min	410 ± 97	100 ± 39	24%
Rat MK-801 Set Shift	3 mg/kg po	150 min	452 ± 60	132 ± 17	29%
	10 mg/kg po	150 min	1,860 ± 507	533 ± 111	29%
	30 mg/kg po	150 min	7,255 ± 573	2,216 ± 36	31%
Mouse MK-801 Y Maze	0.03 mg/kg sc	60 min	5 ± 0.4	ND	ND
	0.10 mg/kg sc	60 min	21 ± 2.4	ND	ND
	0.30 mg/kg sc	60 min	94 ± 19	ND	ND
	1 mg/kg sc	60 min	254 ± 32	ND	ND
	3 mg/kg sc	60 min	774 ± 282	ND	ND
	10 mg/kg sc	60 min	2,982 ± 390	ND	ND
Rat N40 Gating	0.30 mg/kg sc	30 min	81 ± 8	21 ± 6	26%
	0.56 mg/kg sc	30 min	146 ± 12	42 ± 10	30%
	1 mg/kg sc	30 min	295 ± 27	97 ± 14	33%
	3 mg/kg sc	30 mn	1,360 ± 175	372 ± 33	27%
Rat Neonatal PCP ID/ED	0.10 mg/kg po	150 min	<LLQ	ND	ND
	0.30 mg/kg po	150 min	<LLQ	ND	ND
	1 mg/kg po	150 min	21 ± 9	ND	ND
	3 mg/kg po	150 min	225 ± 50	ND	ND
Rat MMN	0.01 mg/kg sc	30 min	2.4 ± 0.1	ND	ND
	0.03 mg/kg sc	30 min	23 ± 29	13	57%
	0.10 mg/kg sc	30 min	24 ± 0.6	ND	ND
	0.30 mg/kg sc	30 min	75 ± 6	13 ± 0.65	17%
	1 mg/kg sc	30 min	398 ± 139	74 ± 18	19%
	3 mg/kg sc	30 min	1,974 ± 272	378 ± 161	19%

LLQ: lower limit of quantification; ND: not determined.
