# Supplementary material for: The Novel, Nicotinic Alpha7 Receptor Partial Agonist, BMS-933043, Improves Cognition and Sensory Processing in Preclinical Models of Schizophrenia
Source: PLoS One. 2016 Jul 28;11(7):e0159996. doi: 10.1371/journal.pone.0159996 (PMC4965148; doi:10.1371/journal.pone.0159996)
Supplement: S4 Table — (PDF) [file pone.0159996.s025.pdf]

**S4 Table. Plasma and brain concentrations of NS-6740 determined after subcutaneous dosing in mice.**

| Dose | Plasma (nM)<br>Mean ± SD | Brain (nM)<br>Mean ± SD | Brain/Plasma<br>Ratio |
|---|---|---|---|
| 0.3 mg/kg | 37 ± 2 | 316 ± 43 | 8.54 |
| 1 mg/kg | 130 ± 7 | 1,125 ± 66 | 8.65 |
| 3 mg/kg | 544 ± 214 | 3,998 ± 273 | 7.35 |
| 10 mg/kg | 2,083 ± 426 | 17,725 ± 1,928 | 8.51 |
